# NAD^+^ Metabolism and Mitochondrial Activity in the Aged Oocyte: Focus on the Effects of NAMPT Stimulation

**DOI:** 10.14336/AD.2024.0241

**Published:** 2024-09-06

**Authors:** Giovanna Di Emidio, Teresa Vergara, Fani Konstantinidou, Irene Flati, Liborio Stuppia, Paolo Giovanni Artini, Valentina Gatta, Stefano Falone, Carla Tatone

**Affiliations:** ^1^Department of Life, Health and Environmental Sciences, University of L’Aquila, 67100 L’Aquila, Italy.; ^2^Department of Psychological Health and Territorial Sciences, School of Medicine and Health Sciences, "G. d'Annunzio" University of Chieti-Pescara, 66100 Chieti, Italy.; ^3^Unit of Molecular Genetics, Center for Advanced Studies and Technology (CAST), "G. d'Annunzio" University of Chieti-Pescara, 66100 Chieti, Italy.; ^4^Department of Biotechnological and Applied Clinical Sciences, University of L’Aquila, 67100 L’Aquila, Italy.; ^5^Department of Obstetrics and Gynecology “P. Fioretti”, University of Pisa, 56126 Pisa, Italy.

**Keywords:** oocyte aging, NAD^+^ metabolism, mitochondrial bioenergetics, P7C3, SIRT1, NAMPT

## Abstract

The ovary experiences an age-dependent decline starting during the fourth decade of life. Ovarian aging is the predominant factor driving female reproductive aging. Modern trend to postpone childbearing age contributes to reduced fertility and natality worldwide. Recently, the beneficial role of NAD^+^ precursors on the maintenance of oocyte competence and female fertility affected by aging has emerged. Nevertheless, age-related changes in NAD^+^ regulatory network have not been investigated so far. In this context, our goal was to investigate changes induced by the aging process in the expression level of genes participating in NAD^+^ biosynthetic and NAD^+^ consuming pathways and in the cellular bioenergetics in the mouse oocyte. From Ingenuity Pathway Analysis (IPA) it emerged that aging caused the downregulation of all cellular pathways for NAD^+^ synthesis (Kynurenine pathway, Preiss-Handler pathway and NAD^+^ salvage pathway) and deeply influenced the activity of NAD^+^-dependent enzymes, i.e. PARPs and SIRTs, with effects on many cellular functions including compromised ROS detoxification. Considering that NAMPT, the rate-limiting enzyme of NAD^+^ salvage pathway, was deregulated, aged oocytes were matured in the presence of P7C3, NAMPT activator. P7C3 improved spindle assembly and mitochondrial bioenergetics and reduced mitochondrial proton leak. Moreover, P7C3 influenced gene expression of NAD^+^ regulatory network, with Sirt1 as the central node of IPA-interfered target gene network. Finally, P7C3 effectively counteracted oocyte alterations induced by exposure to oxidative stress. Our study contributes to establish effective NAD^+^ boosting interventions to alleviate the effects of advanced maternal age on fertility and explore their potential in redox-related fertility disorders.

## INTRODUCTION

Nicotinamide adenine dinucleotide (NAD) is a multifunctional metabolite in living cells with a prominent role in redox reactions, cell energy production, cellular metabolism, and survival. Cellular NAD exists in two forms, oxidized (NAD^+^) and reduced (NADH) [1]. NAD^+^ is responsible for accepting high-energy electrons and carrying them to the electron transport chain (ETC) within mitochondria to drive the biosynthesis of adenosine triphosphate (ATP). The NAD^+^/NADH ratio reflects the metabolic balance of the cell in generating ATP energy and is critical for normal cell function and viability. By acting as a co-substrate, NAD^+^ can directly and indirectly impact a number of cellular functions associated with metabolic bioenergetics [2].

**Figure 1.**
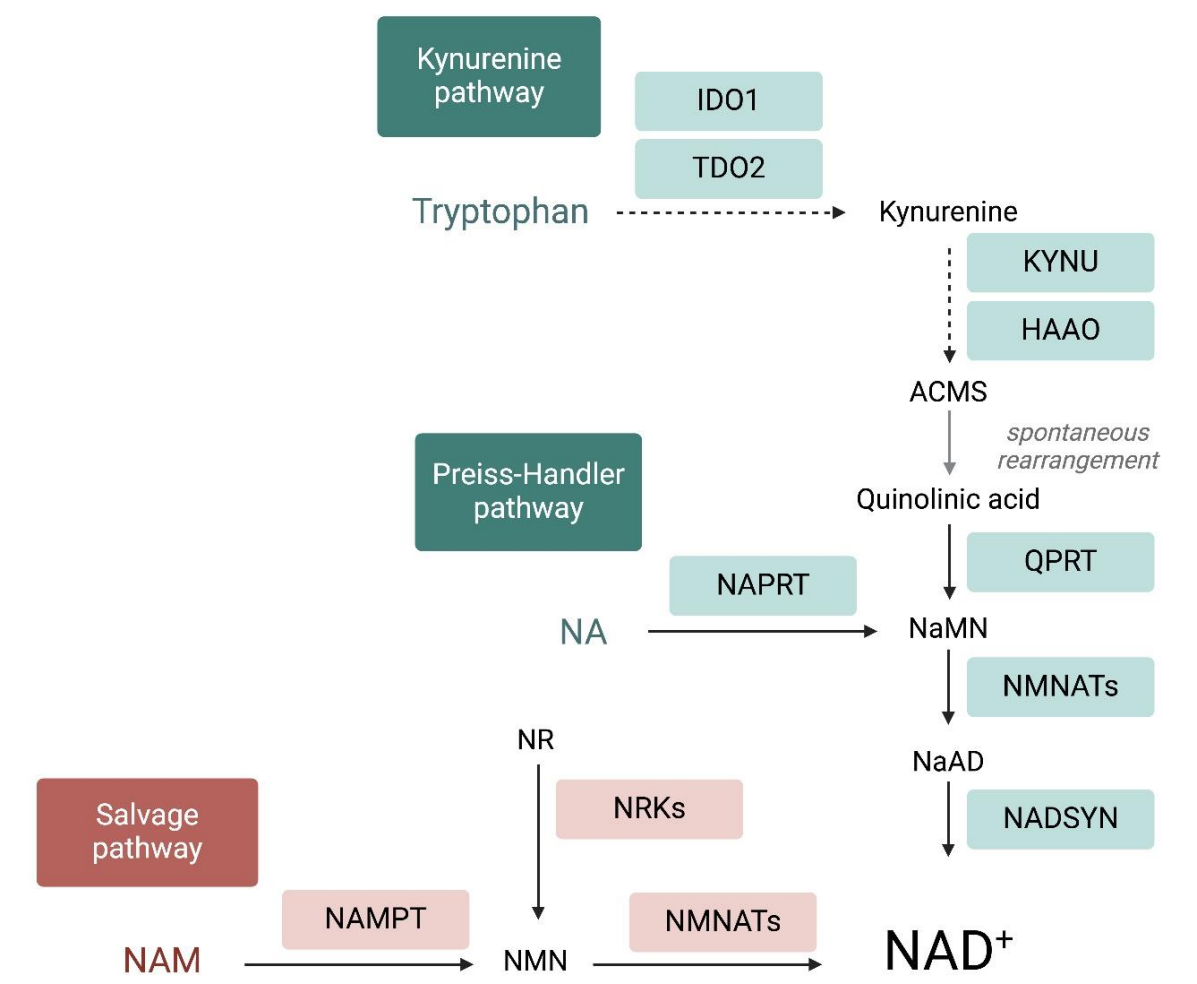
**Intracellular pathways of NAD^+^ production**. Mammalian cells can synthesize NAD^+^ *de novo* from tryptophan by the kynurenine pathway or from NA by the Preiss-Handler pathway, while most NAD^+^ is recycled via salvage pathways by NAMPT from NAM, a by-production of NAD^+^-consuming reactions. Abbreviations: ACMS: α-amino-β-carboxymuconate-ε-semialdehyde; HAAO, 3-Hydroxyanthranilic Acid Dioxygenase; IDOs, indoleamine 2,3-dioxygenase; KYNU, Kynureninase; NA, nicotinic acid; NaAD, Nicotinic acid adenine dinucleotide; NADKs, NAD^+^ kinases; NADSYN, NAD synthase; NAM, nicotinamide; NaMN,nicotinate mononucleotide; NAPRT, nicotinic acid phosphoribosyltransferase; NMN, nicotinamide mononucleotide; NMNATs, nicotinamide mononucleotide adenylyl transferases; NR, nicotinamide riboside; NRK, Nicotinamide Riboside Kinase; QPRT, quinolinate phosphoribosyl-transferase; TDO, tryptophan 2,3-dioxygenase. The image was generated by Biorender.

The NAD^+^ pool is set by a critical balance between NAD^+^ biosynthetic and NAD^+^ consuming pathways. Generally, NAD^+^ supply is regulated through biosynthesis from the precursors delivered with the diet. As reported in Fig. 1, NAD^+^ can be generated from amino acid L-tryptophan through the kynurenine pathway (*de novo* synthesis). Another source for NAD^+^ is nicotinic acid (NA, vitamin B3), a precursor that is converted to nicotinic acid mononucleotide (NaMN) and subsequently to nicotinic acid adenine dinucleotide phosphate (NaAD) and NAD^+^ (Preiss-Handler pathway). A further process responsible for NAD^+^ production is the NAD^+^ salvage pathway in which nicotinamide mononucleotide (NMN) is either produced from nicotinamide riboside (NR) by NR kinase (NRK) or from nicotinamide (NAM) by nicotinamide phosphoribosyltransferase (NAMPT, Fig. 1). NAD^+^ can be also formed after nucleotide transhydrogenase (NNT)-dependent transfer of a hydrogen from NADH to NADP^+^ [3]. For mammalian cells, NAD^+^ synthesis is achieved predominantly via the NAMPT-mediated NAD^+^ salvage pathway [4, 5].

NAD^+^ consuming pathways include enzymes that utilize NAD^+^ as a cofactor to control gene expression, stress response, DNA repair, apoptosis and mitochondrial biogenesis, such as poly- (ADP-ribose) polymerases (PARPs), sirtuins (SIRTs), CD73, and CD38 [4, 5].

Cumulating evidence has suggested that NAD^+^ deficiency is a common pathological factor of a number of diseases and aging [6]. Boosting NAD^+^ levels through exogenous administration of precursors has the potential to prevent or alleviate a wide range of diseases, such as metabolic and age-related disorders [4, 5]. Interestingly, maternal deficiency in NAD^+^ production has been linked to an increase in spontaneous abortion and congenital defects in mice and women [7]. Mutant knockout mice exhibiting NAD^+^ deficiency and fed a diet without NAD^+^ precursors exhibited higher rates of miscarriage and congenital malformations in surviving embryos. These effects were mitigated by supplementing maternal diets with niacin (vitamin B3) [7]. This finding has stimulated research on NAD^+^-elevating agents as possible tools to improve female fertility in a range of species [8-13].

Female fertility is strongly affected by the aging process. The ovary is the first organ to experience an age-dependent decline starting during the fourth decade of life. Ovarian aging is the predominant factor driving female reproductive aging, associated with deleterious effects on fertility, but also on health and well-being. Thus, maternal age plays a significant part in determining oocyte quality and lowers the chance of pregnancy and live birth [14-16]. Oocyte aging derives from the prolonged stay of the oocytes of the primordial follicle pool in a resting phase as well as to its exposure to the aged ovarian and follicular microenvironment during growth and final maturation [14-16]. This decline has largely been attributed to alterations in mitochondrial functions leading to oxidative stress, responsible for mitochondrial damage, suboptimal intracellular energy levels, calcium disturbance, and meiotic spindle alterations. This contributes to increased aneuploidy along with DNA damage, loss of chromosomal cohesion, spindle assembly checkpoint dysfunction, meiotic recombination errors, and telomere attrition, along with altered mitochondrial dynamics, dysmorphic smooth endoplasmic reticulum, calcium disturbance, and cytoskeletal alterations. Aged oocytes seem therefore to mature in an altered microenvironment, with changes in metabolites, RNAs, proteins, and lipids. Overall, a more comprehensive understanding of the mechanisms implicated in the loss of oocyte quality will allow the establishment of emerging biomarkers and potential anti-aging strategies.

Of note, knockout of key enzymes involved in *de novo* NAD^+^ biosynthesis results in a reproductively aging-like phenotype with the production of oocytes with reduced developmental potential, characterized by defective spindle and dysfunctional mitochondria [13]. Similar effects are observed in mouse oocytes by knockdown of the NAD^+^ biosynthetic enzyme NAMPT, with subsequent reduction of NAD^+^ and ATP levels, spindle abnormalities and compromised asymmetry of meiotic division [17]. Indeed, NAD^+^ levels are decreased in oocytes and ovaries of reproductively aged mice [8, 9, 12, 13, 18], thus supporting the notion that ovarian aging may be associated with impaired NAD^+^ metabolism in the oocyte. There is substantial evidence that supplementing aging mice with the NAD^+^ precursors NR or NMN promotes rejuvenating effects on reproductive function, such as increasing the number of ovulated oocytes, meiotic competency, fertilization and live births associated with effects on the redox state and mitochondria [8, 9, 19].

However, beyond the observation of both reduced NAD^+^ levels in female gametes during aging and efficacy of some approaches based on NAD^+^ boosting as ovarian anti-aging intervention in animal models, deregulated pathways underlying altered NAD^+^ metabolism with oocyte aging remain very poorly investigated.

In this context, the aim of the study was to establish whether aging affects the expression of genes strictly involved in NAD^+^ biosynthetic and NAD^+^ consuming pathways in mammalian oocytes. Then, the effect of stimulation of the NAD^+^ salvage pathway by P7C3 was studied in terms of capacity to improve *in vitro* maturation, mitochondrial bioenergetics and gene regulation of NAD^+^ metabolism in aged oocytes.

## MATERIALS AND METHODS

### Oocyte collection and treatment

Oocytes were isolated from young (4-8 weeks) and reproductively aged (40-52 weeks) CD-1 female mice (Charles River s.r.l. Calco, Italy). Mice were maintained in a temperature-controlled environment under a 12 h light/dark cycle (07:00-19:00) and free access to feed and water *ad libitum*. All the experiments were carried out in conformity with national and international laws and policies. The project was approved by the Italian Ministry of Health and the Internal Committee of the University of L’Aquila (Authorization n° 329/2022-PR).

Young and reproductively aged mice were superovulated by intraperitoneal injection of 10 IU of PMSG (Folligon; Intervet-International, Boxmeer, Holland) and 10 IU of hCG (Gonasi HP 2000 U.I.; Serono, Roma, Italy) 48 h apart. After 15 h, mice were killed by cervical dislocation and oviducts were removed. Cumulus masses were released into the M2 medium (M7167, Sigma-Aldrich, St. Louis, MO, USA) and oocytes arrested at metaphase II stage (MII oocytes) were isolated after a brief exposure to 0.3 mg/ml hyaluronidase (H3506, Sigma-Aldrich).

Ovarian immature oocytes at the germinal vesicle stage (GV) were collected from PMSG-primed mice 48 h after 10 U.I. PMSG. Meiotic block at GV stage was achieved by addition of 0.5 μM cilostamide (231085, Sigma-Aldrich) in the culture media, M2 or M16 (M7292, Sigma-Aldrich), according to each procedure [19]. After collection, young oocytes were randomly assigned to control, H_2_O_2_ or H_2_O_2_ + P7C3 group; aged oocytes were randomly assigned to plain medium or supplemented with P7C3.

Experiments on *in vitro* maturation (IVM) were performed by 16 h *in vitro* culture of GV oocytes from PMSG-primed mice in M2 medium in a non-CO_2_ humidified incubator at 37 °C. To induce oxidative stress, GV oocytes were exposed to 100 μM H_2_O_2_ for 10 min or 200 μM H_2_O_2_ for 15 min prior to 16 h IVM [19] in presence or absence of 5 μM P7C3, at 37 °C in a non-CO_2_ humidified incubator. Numbers of oocytes that emitted the first polar body (MII) were recorded.

After treatment, oocytes were immediately processed for further analysis. A list of all experimental classes is resumed in Table 1.

**Table 1:** List of oocyte experimental classes and oocyte treatments.

Experimental class	Mouse age and hormonal stimulation	Pre-Treatment	Insult	IVM condition
***MII oocytes***				
**Young MII**	Young, PMSG+hCG primed	-	-	-
**Aged MII**	Aged, PMSG+hCG primed	-	-	-
***GV oocytes***				
**Young GV**	Young, PMSG primed	0.5 μM cilostamide	-	-
**Aged GV**	Aged, PMSG primed	0.5 μM cilostamide	-	-
***IVM oocytes (In vitro maturation of GV oocytes for 16 h)***				
**Young IVM**	Young, PMSG primed	-	-	M2 medium
**Aged IVM**	Aged, PMSG primed	-	-	M2 medium
**Aged IVM P7C3**	Aged, PMSG primed	-	-	1-5 μM P7C3
**Young IVM H_2_O_2_**	Young, PMSG primed	-	100 μM H_2_O_2_ for 10’	M2 medium
**Young IVM H_2_O_2_ P7C3**	Young, PMSG primed	-	100 μM H_2_O_2_ for 10’	5 μM P7C3

Degenerated or fragmented cells were discarded, and healthy oocytes were pooled and randomized before distribution into the experimental groups. Each experiment was performed three times and at least 25 oocytes per group were employed in each replicate.

### NAD^+^ signalling pathway quantitative real-time PCR array

Pools of 25 oocytes were conserved in lysis buffer at -80 °C up to moment of manual extraction. Total RNA was extracted using the NucleoSpin miRNA kit (740955.50, Macherey-Nagel, Milan, Italy) according to manufacturer’s instructions. Quantity and quality of total RNA was assessed by microvolume UV-vis spectrophotometer NanoPhotometer (Implen, GmbH, Munich, Germany). Total RNA (1 μg) was used for cDNA synthesis in a 20 μL reaction through the high-capacity cDNA reverse transcription kit (4368813; Applied Biosystems, Foster City, CA, USA) under the following conditions: 25 °C for 10 min, 37 °C for 120 min, 85 °C for 5 min and a final cooling temperature at 4 °C. Each cDNA was then analysed in duplicate by employing a NAD Metabolism M96 Predesigned 96-well panel (Mouse) (10029342, Bio-Rad Laboratories, CA, USA) (Supplementary Fig. 1), containing 40 genes of the NAD pathway, 3 housekeeping genes, 5 reaction control probes. The 2X SsoAdvanced Universal SYBR Green Supermix (Bio-Rad Laboratories, CA, USA) was used to amplify targets in an ABI 7900HT sequencing detection system (1725271, Life Technologies, Carlsbad, CA, USA). Upon completion of the reaction cycles, melting curves were obtained by heating the reactions from 60 °C to 95 °C. The specificity of the primers was confirmed by the presence of a single peak in the melt curve generated for all gene targets.

### PCR data analysis

Raw data were analysed by the DataAssist software (ThermoFisher Scientific, Waltham, MA, USA). Analyses were carried out normalization based on housekeeping genes. Only genes showing no outlier replicates and a maximum allowable Ct value of 38 in the murine oocytes were included in the analysis. A gene was considered differentially expressed in aged oocytes versus young oocytes when showing a fold change >1.4 or <0.7 and a *p*-value < 0.05 (One-Way ANOVA). *p*-values were adjusted using Benjamini-Hochberg FDR correction test.

### Ingenuity Pathway Analysis (IPA)

The identified up- and down-regulated genes were analyzed by IPA software (Ingenuity Systems, Redwood City, CA) to find out the biological functions and the functional networks they are involved in. IPA functional and network analyses were conducted as previously reported [20].

### Immunofluorescence

For meiotic spindle analysis, oocytes were fixed for 30 min at 37 °C in a microtubule-stabilizing buffer, containing 2% formaldehyde (104003, Sigma-Aldrich), 0.1% Triton X-100 (112298, Sigma-Aldrich), 1 µM taxol (T1912, Sigma-Aldrich), 10 IU/ml aprotinin (A1153, Sigma-Aldrich) and 50% deuterium oxide (293040, Sigma-Aldrich). After overnight blocking with a phosphate-buffered saline (PBS, BCBD7947, Sigma-Aldrich) supplemented with 1% BSA (A-3311, Sigma-Aldrich), 0.2% powdered milk (5C015681, Sigma-Aldrich), 2% normal goat serum (G9023, Sigma-Aldrich), 0.1 M glycine (G8898, Sigma-Aldrich), 0.01% Triton X-100 (112298, Sigma-Aldrich), oocytes were incubated in the presence of a mouse monoclonal anti-α-tubulin (T9026, 1:150; Sigma-Aldrich) followed by donkey anti-mouse IgG antibody DyLight® 594 conjugated (A90-137D4, 1:500; Bethyl Laboratories, Montgomery, USA) for 1 h at 37 °C each. Oocytes were labelled with 1 µg/ml Hoechst 33342 (14533, Sigma-Aldrich) in blocking solution for 10 min at room temperature and mounted on slides. Oocytes were analyzed using a fluorescence microscope (AxioPlan 2, Zeiss; 40x objective) with digital images collected with Leica DFC350 FX camera interfaced with IM500 Leica software.

**Figure 2.**
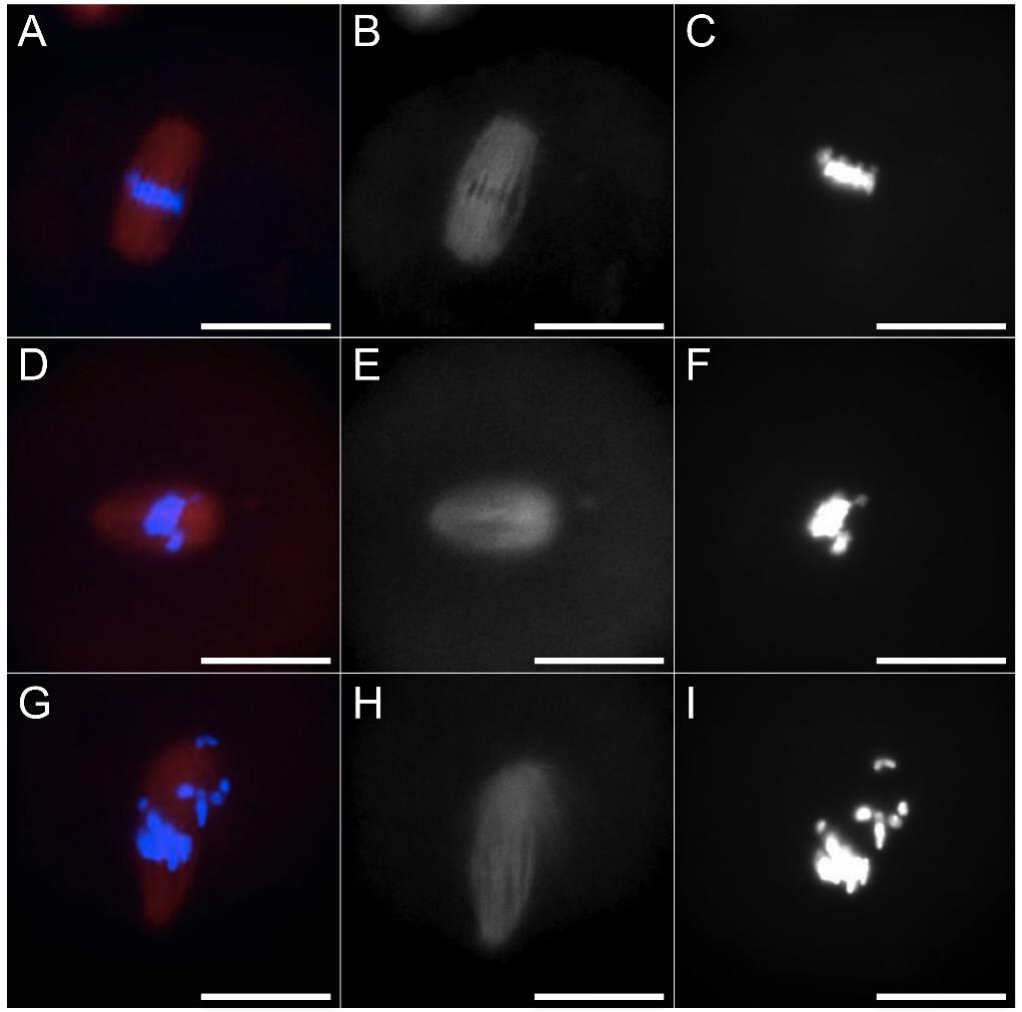
**Representative images of MII spindle and chromosome configuration in oocytes showing polar body after IVM**. Meiotic spindle was labeled by mouse anti -tubulin primary antibody and secondary antibody conjugated with DyLight® 594 (red); chromosomes were stained by Hoechst 33342 (blue). Oocytes were classified as normal **(A)**, slightly aberrant **(D)** or aberrant **(G)**. Spindle was classified as normal **(B)**, slightly aberrant **(E)**, or aberrant **(H)**. Chromosomes were classified as normal **(C)**, slightly aberrant **(F)**, or aberrant **(I)**. Scale bars: 20 μm.

The oocytes underwent classification into the following groups: (i) normal, characterized by the proper assembly of a bipolar mitotic spindle and accurate alignment of chromosomes; (ii) slightly aberrant, characterized by a meiotic spindle displaying slight disorganization of microtubules or exhibiting a slightly abnormal structure, along with a minor dispersion of chromosomes, with a maximum of four scattered chromosomes; (iii) aberrant, characterized by a mitotic spindle that was completely disorganized, abnormal, or absent, and/or by total disorganization of DNA on the MII plate, or the presence of non-condensed chromosomes (Fig. 2) [21, 22].

For NAMPT and SIRT1 analysis, oocytes were subjected to zona pellucida removal by pronase (53702; Sigma-Aldrich) for 7 min. Subsequently, dezoned oocytes were fixed, washed with PBS-PVP, permeabilized with 0.1 % of Triton X-100 in PBS-PVP for 5 min. Oocytes were blocked by exposure to 0.01 % tween-20 (P1379, Sigma-Aldrich) and 0.1 % BSA in PBS-PVP (blocking solution), and labelled by first antibody rabbit anti-SIRT1 (ab189494, Abcam, Cambridge, UK), or mouse anti-NAMPT (MA5-50951, Invitrogen, Waltham, Massachusetts, USA) and second antibody anti-rabbit IgG Alexa Fluor™ 488 (A-11008, Invitrogen) or donkey anti-mouse IgG antibody DyLight® 594 conjugated, respectively. Oocytes chromosomes were labelled with 1 µg/ml Hoechst 33342 in blocking solution for 10 min at room temperature, mounted on slides and analysed under confocal microscope (TCS SP5 II, Leica Microsystems, Germany). In negative control oocytes, the primary antibody was omitted. In order to enable experiment comparison, the settings were maintained in all the experiments. NAMPT and SIRT1 signal was quantified by Image J 1.52a software (National Institutes of Health).

### Application of Seahorse XFp to measure oxygen consumption in oocytes and embryos

Sensor containing Seahorse fluxpacks (103798; Agilent Technology, Santa Clara CA; USA) were incubated overnight at 37 °C in a non-CO_2_ humidified incubator and calibrated according to manufacturer’s instructions. Oocytes were analyzed using a specialized protocol involving a 15 min equilibration period upon loading the cell plate, and alternating between a 3 min measurement period and a 1 min re-equilibration period, according to [22]. Plate specific ‘blank’ cell-free wells containing culture medium were used to account for environmental changes and flux of oxygen in the absence of cells. Inhibitors were dissolved in 100% DMSO (D8779; Sigma-Aldrich) or medium and diluted in warmed analysis media within 30’ from the beginning of the assay. Serial injections of 0.5 μM oligomycin, 100 μM 2,4-DNP (D198501, Sigma-Aldrich), and 1 μM antimycin A and rotenone (A/R), were used to analyse oocyte mitochondrial bioenergetics [22]. Briefly, oligomycin was used to inhibit ATP synthase to identify ATP-linked oxygen consumption. 2,4-DNP was used as an uncoupling compound that collapses the inner mitochondrial membrane gradient, thus allowing the study of ETC functioning at maximal capacity. Finally, in order to correct oxygen consumption from non-mitochondrial oxidases, inhibitors of respiratory complex I and III rotenone and antimycin A, respectively, are added. “Proton leak,” can be obtained by subtracting non-mitochondrial respiration from the value of ATP-linked respiration; and spare respiratory capacity, by subtracting basal respiration from the value of maximal respiration. This allowed us to analyze and compare basal respiration (baseline OCR prior to inhibitor injection), proton leak, spare respiratory capacity and ATP production in the different experimental groups.

The analysis was performed on pools of 5-8 oocytes. Each measure was performed at least on 3 replicates. Wave software (Agilent Technologies) was used to determine oxygen consumption, which was expressed as pmol O_2_/min/well. This value was normalized to numbers of oocytes per well.

### ATP assay

Measurements of cytosolic ATP levels were performed according to Cell Titer-Glo ATP assay kit (G9241; Promega Ltd, Southampton, UK), as recommended by the manufacturer. Briefly, pools of 3-7 oocytes were placed in M2 medium in 96-well white bottom plates (17122001; Corning, New York, USA). The reaction mix induced cell lysis and contained a proprietary thermostable luciferase, which generates a luminescent signal that is proportional to the amount of ATP present. The light signals were taken as the steady state values. Light was recorded using a Viktor luminometer (PerkinElmer, Waltham, MA, USA). The signals were calibrated with a series of dilutions of ATP (A7699, Sigma-Aldrich) ranging from 0-50 nM and normalized to the number of oocytes in each well.

### NAD^+^, NADH and total NAD quantification

The measurement of NAD^+^, NADH and total NAD was evaluated by NAD/NADH-Glo™ bioluminescence Assay kit (G9071, Promega), according to manufacturer’s instructions. Briefly, pools of at least 25 oocytes were placed in 96-well white bottom plates in 50 µL PBS. To induce cell lysis 50 µL of 1% DTAB (D8638, Sigma Aldrich) in 0.2 N NaOH (203387B, Santa Cruz Biotechnology Inc., Dallas TX, USA) were added. The 100 µL solution containing lysated oocytes was splitted in two wells for NAD^+^ and NADH quantification. For NAD^+^ quantification, 25 µL of 0.4 N HCl (186985; PanReac AppliChem, Darmstadt, Germany) were added to 50 µL of lysated oocytes and incubated for 15 min at 60 °C, followed by 10 min at room temperature. Then, 25 µL of 0.5 M TRIZMA base (A2264; PanReach AppliChem) were added. For NADH quantification, 50 µL of a solution of 0.4 N HCl and 0.5 M TRIZMA base were added to 50 µL of lysated oocytes.

Then, NAD^+^/NADH detection reagent containing luciferin was added to each well. The light signals were taken as the steady state values. Light was recorded using a Viktor luminometer (PerkinElmer, Waltham, MA, USA). The signals were calibrated with a series of dilutions of 0-50 nM NAD^+^ (N8285, Sigma-Aldrich) and 0-50 nM NADH (N6660, Sigma-Aldrich) and normalized to the number of oocytes in each well. Total NAD was obtained by summing NAD^+^ and NADH oocyte content.

### NAD(P)H autofluorescence

Live oocytes were transferred in M2 medium on a modified slide, with a culture chamber, and quickly observed under confocal microscope. Oocyte auto-fluorescence was imaged as previously described by [23, 24]. Briefly, blue autofluorescence emitted by the pyridine nucleotides NADH and NADPH in their reduced form was excited with UV light (405 nm) on confocal laser scanning microscopy and emission was collected using a 435-485 nm bandpass filter. In order to enable experiment comparison, the settings were maintained in all the experiments. Autofluorescence signal was quantified by Image J 1.52a software (National Institutes of Health).

### Statistical analysis

Values are reported as means ± SEMs. All data were tested for normal distribution by Shapiro-Wilk test. Statistical differences were assessed by t-test, Mann-Whitney test, square Chi test, one way ANOVA or Kruskal-Wallis test. Analyses were performed using the SigmaPlot 12 (Systat Software Inc., San Jose, CA, USA) and GraphPad Prism 8.0.1 (GraphPad Software, Boston, MA USA). A p value of <0.05 was considered statistically significant.

## RESULTS

### Aging affects NAD^+^ metabolism in MII oocytes: IPA-inferred functional and network analysis of differentially expressed genes involved in NAD^+^ metabolism

**Figure 3.**
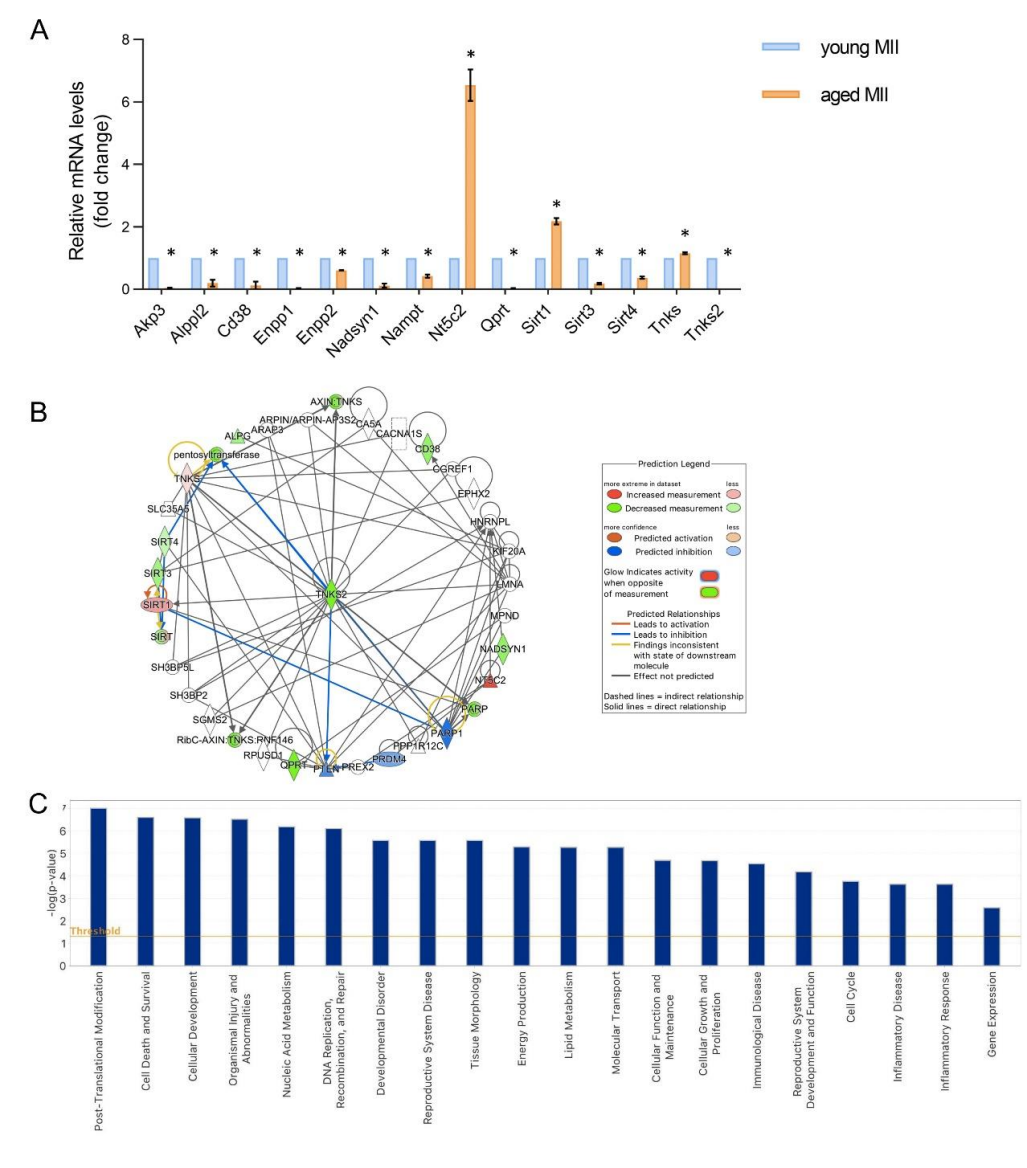
**Differentially expressed genes involved in NAD^+^ metabolism in young and aged MII oocytes and Ingenuity pathway analysis (IPA)-generated functional analysis. (A)** Histograms of significant mean fold change values for all differentially expressed genes in physiological aged oocytes compared to young controls. Pools of 25 oocytes isolated from 3-6 mice were employed. The experiment was repeated three times. Statistical analysis by paired t-test: ^*^p<0.05. **(B)** IPA-interfered target gene network for NAD^+^ metabolism of physiologically aged MII oocytes compared to controls. Tnks2 gene is the central node of IPA-interfered target gene network for NAD^+^ metabolism of physiologically aged oocytes in comparison to young oocytes. In red the up-regulated genes, while in green the down-regulated ones. Blue arrow lines indicate a predicted inhibition. **(C)** The bar-chart is generated based on a -log (*p*-value) threshold of 0.05 and indicates the main significant biological functions regulated by our gene dataset in aged oocytes.

Statistical analysis of the expression of genes related to NAD^+^ biosynthetic and consuming pathways showed a significant down-regulation of 11 genes (Akp3, Alppl2, Cd38, Enpp1, Enpp2, Nadsyn1, Nampt, Qprt, Sirt3, Sirt4 and Tnks2) (p<0.05) in aged MII oocytes compared to young controls. A significant up-regulation of 3 genes, Nt5c2, Sirt1 and Tnks was detected in the same samples (Fig. 3A).

IPA analysis was performed for the 14 differentially expressed transcripts, in order to highlight the main key functions and cellular processes in which they are involved, as well as to generate a mechanistic gene network based on their connectivity and enrichment statistics. A Fisher’s exact test was used to generate the network score, based on the number and size of eligible genes and the total number of genes that could be included in the network.

By applying a -log (p-value) threshold of 0.05, 21 representative functions were evidenced as shown in Fig. 3C. Among them, the most relevant ones were the following: post-translational modifications, cell death and survival, cellular development, organismal injury and abnormalities, nucleic acid metabolism, DNA replication, recombination and repair, developmental disorders, reproductive system disease and tissue morphology, energy production, lipid metabolism and molecular transport (Fig. 3C).

Each box of the functional IPA heatmap represents a biological class, which is split into secondary functions. The size of the box depends on the significant IPA-inferred p-values, while the color on IPA-generated z-score. The sign of the calculated z-score reflects the overall predicted activation state of the biological function. In general, z-scores greater than 2 or smaller than -2 can be considered significant. (Supplementary Fig. 2).

Moreover, an IPA network analysis was executed by predicting the drivers of the modulated gene expression based on a value calculated by the IPA z-score algorithm. A top IPA-inferred network was generated with a score ranging from 29 to 7, as provided in Fig. 3B, and is centered around the key node gene Tnks2, a member of the PARP family.

**Figure 4.**
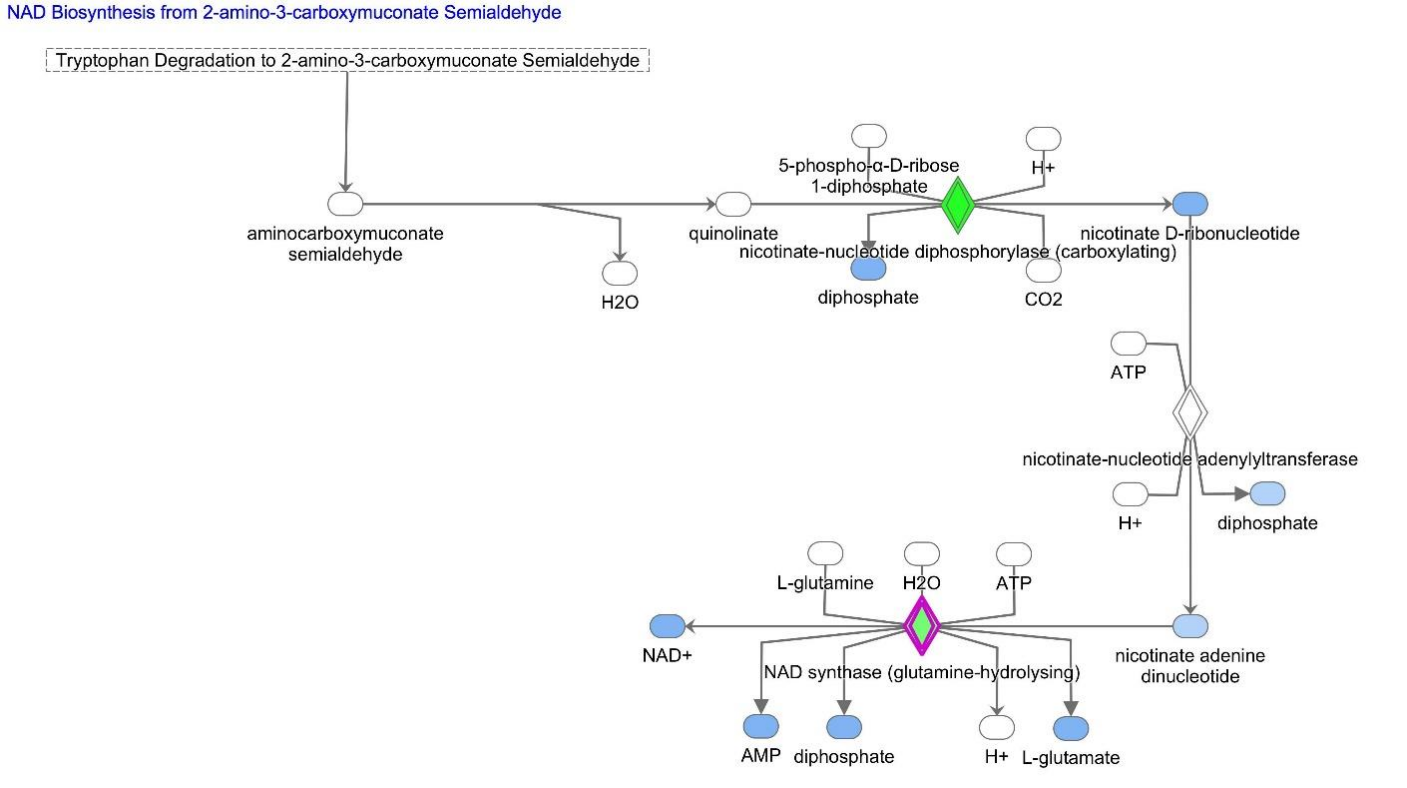
**Schematic depiction of the NAD^+^ biosynthetic pathways with genes significantly down regulated in aged oocytes**. In green are indicated products genes significantly down regulated in aged oocytes in comparison to young oocytes. In blue are indicated products that are predicted to be reduced as a consequence of reduced enzymatic activity. The canonical pathways were analyzed using QIAGEN’s Ingenuity Pathway Analysis (IPA; QIAGEN Inc., www.qiagenbioinformatics.com/products/ingenuity-pathway-analysis). Differentially expressed genes in aged oocytes were subjected to IPA analysis, and significant canonical pathways were identified at p<0.05. The above-identified pathways demonstrate that genes differentially expressed with aging influence NAD^+^ biosynthesis leading to a reduction of NAD^+^ availability.

**Figure 5.**
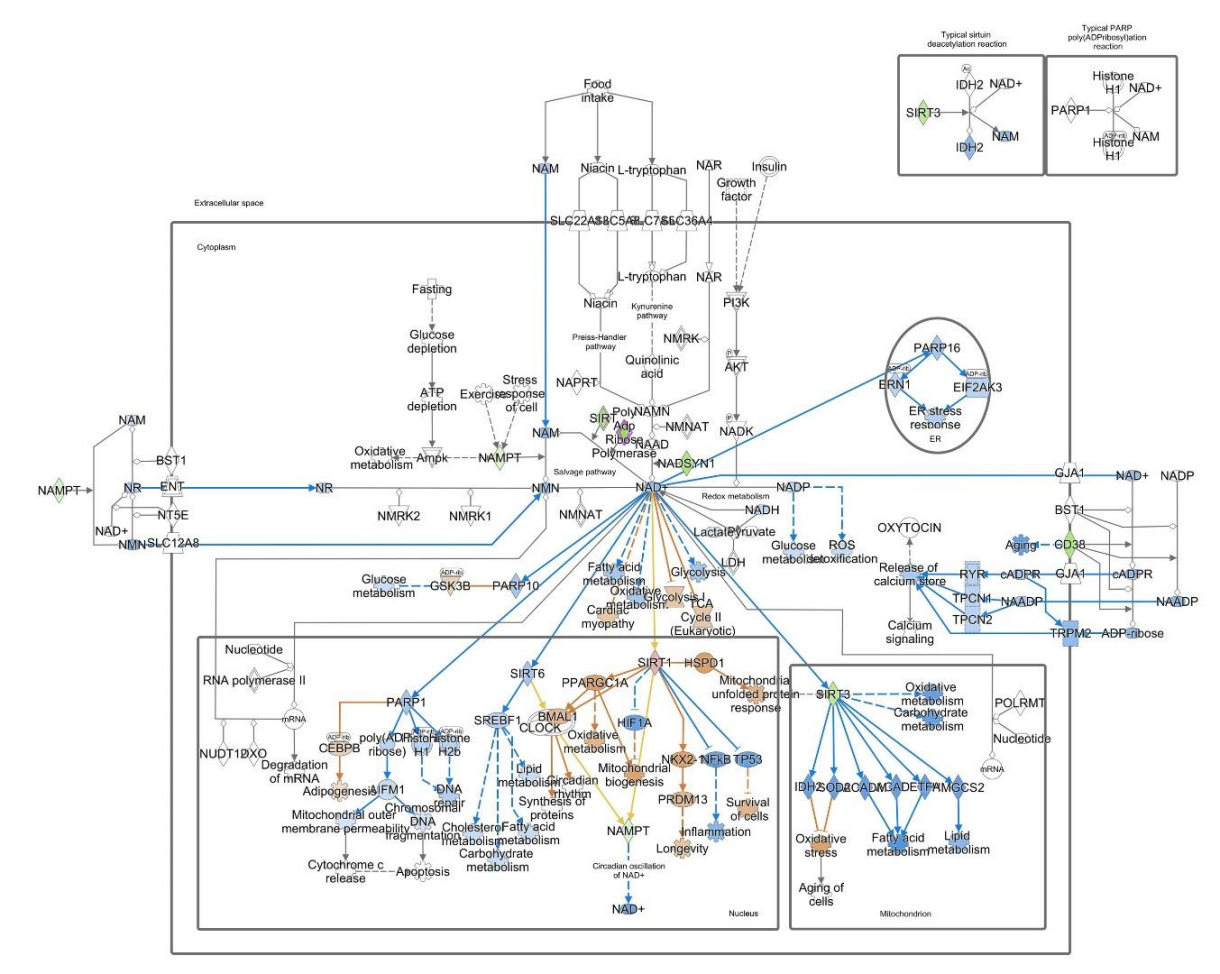
**NAD^+^-related metabolic pathways influenced by oocyte aging generated by Ingenuity pathway analysis (IPA) analysis**. Signaling pathway diagram highlighting the effects of aging on NAD^+^ metabolism in the mouse MII oocyte. At nucleus level the observed increased level of SIRT1 is connected with predicted increased longevity, reduced inflammation and increased survival, activation of protein unfolded response. It is also predicted the activation of PPARGC1A leading to increased mitochondrial biogenesis; the inhibition of SIRT6 with negative effects on metabolism; the inhibition of PARP1 on DNA repair and mitochondrial integrity. At cytoplasmic level, the observed NAMPT decrease leads to predictive reduction of metabolites and NAD^+^. Predicted decreased NAD^+^ reduction leads to predictive reduction of PARPs and SIRTs in other compartments. Decreased oxidative metabolism and ROS detoxification are also predicted. At mitochondrial level it is observed a decreased activation of SIRT3, while it is predicted the inhibition of oxidative metabolism and carbohydrate metabolism and the activation of oxidative stress, reduced fatty acid metabolism and lipid metabolism. This network was derived from QIAGEN’s Ingenuity Pathway Analysis (IPA; QIAGEN Inc., www.qiagenbioinformatics.com/products/ingenuity-pathway-analysis). NADD: nicotinic acid adenine dinucleotide; NAADP: nicotinic acid adenine dinucleotide phosphate; NAD: nicotinamide adenine dinucleotide; NADP: nicotinamide adenine dinucleotide phosphate; NAM: niacinamide/nicotinamide; NAMN: nicotinic acid d-ribonucleotide; NAR: nicotinic acid d-ribonucleoside; NMN: nicotinamide mononucleotide; NR: nicotinamide ribonucleoside.

Finally, pathway activity analysis was performed to estimate whether canonical pathways may be found activated or inhibited based on the expression or phosphorylation of significantly modulated genes in our dataset. From IPA it emerged that differentially expressed genes cause a down regulation of the two NAD^+^ biosynthetic pathways, that is Kynurenine pathway (or *de novo* NAD^+^ production) and Preiss-Handler pathway (Fig. 4). This condition predicts a reduction of NAD^+^ production, and thus bioavailability, in the aged oocytes (Fig. 4). Moreover, IPA network analysis based on genes differentially expressed with aging revealed that low NAD^+^ availability represents the core of the diagram influencing many cellular functions (Fig. 5). Fig. 5 shows that reduced levels of NAD^+^ may derive also from reduced NAD^+^ salvage pathway. NAD^+^ reduction deeply impacts the activity of NAD^+^-dependent enzymes PARPs and SIRTs and is associated with a condition of decreased oxidative metabolism and reduced ROS detoxification. In the mitochondria, data from dataset report a level of SIRT3, which negatively influences carbohydrate, fatty acids and lipid metabolism and leads to the establishment of oxidative stress. In the nucleus, the observed increased level of SIRT1 could be suggestive of reduced inflammation, increased survival, and activated unfolded protein response (UPR). Activation of PPARGC1A and the inhibition of both SIRT6 and PARP1 could be also predicted, thus leading to hypothesize that an increased mitochondrial biogenesis, dysregulated metabolism and impaired DNA repair may occur, along with loss of mitochondrial integrity.

### NAD content is increased in aged oocytes upon NAMPT stimulation/activation by P7C3

**Figure 6.**
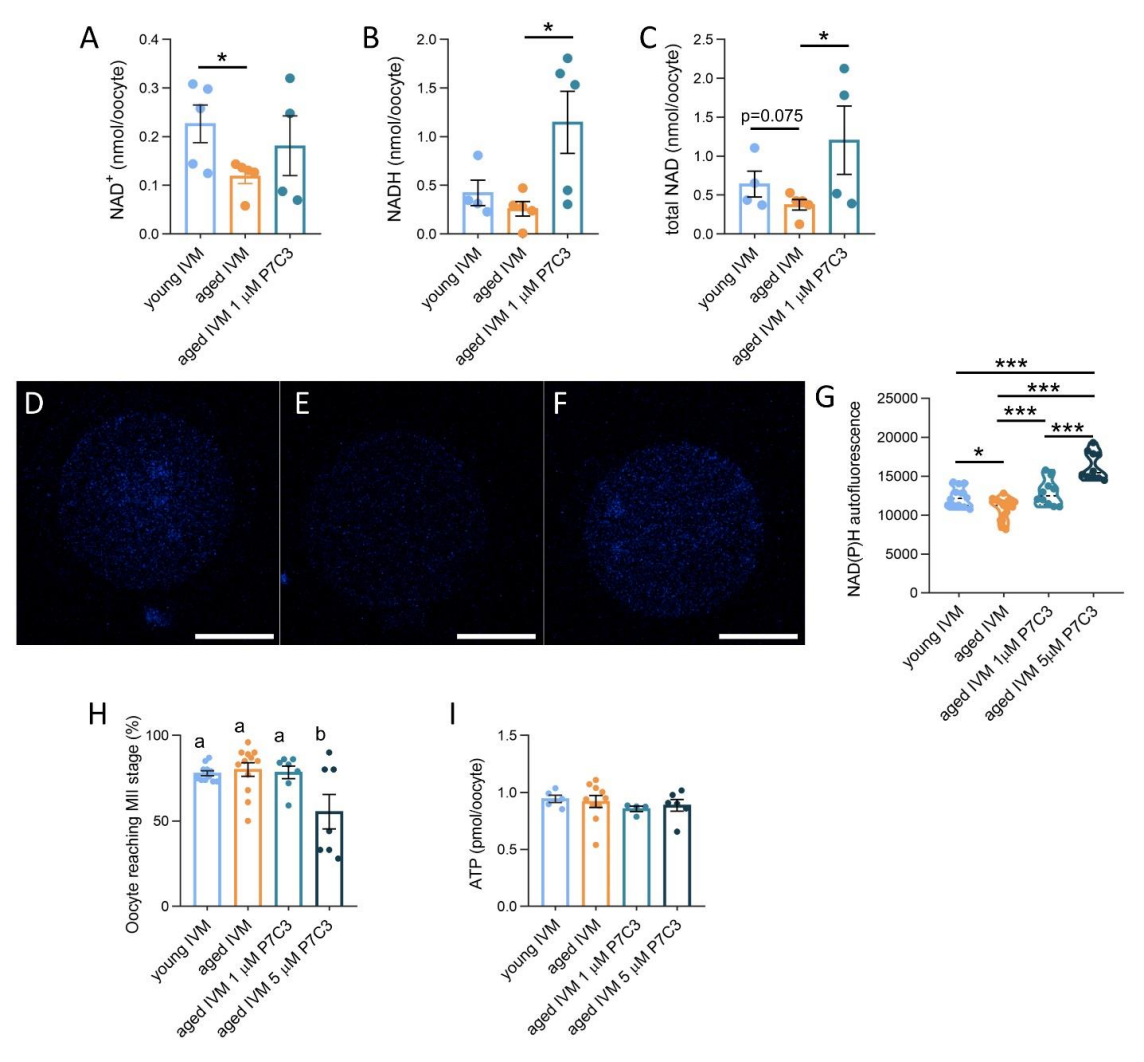
**Effect of NAMPT stimulation by P7C3 on NAD content, IVM rate, ATP production of aged oocytes. (A)** Bioluminescent quantification of NAD^+^ in young, aged and aged oocytes exposed to NAMPT stimulation by P7C3 during IVM. In brackets numbers of pools: young (n=5); aged (n=5); aged 1 µM P7C3 (n=4). Pools of at least 25 oocytes collected from 3-6 mice were employed. Statistical analysis by Mann-Whitney test. *p=0.0476. **(B)** Bioluminescent quantification of NADH in young, aged and aged oocytes exposed to NAMPT stimulation by P7C3 during IVM. In brackets numbers of pools: young (n=4); aged (n=5); aged 1 µM P7C3 (n=5). Pools of at least 25 oocytes collected from 3-6 mice were employed. Statistical analysis by unpaired t-test *p=0.0263. **(C)** Bioluminescent quantification of total NAD in young, aged and aged oocytes exposed to NAMPT stimulation by P7C3 during IVM. In brackets numbers of pools: young (n=4); aged (n=5); aged 1 µM P7C3 (n=4). Pools of at least 25 oocytes collected from 3-6 mice were employed. Statistical analysis by unpaired t-test *p=0.0365. Representative confocal images of autofluorescence to determine NAD(P)H content in young **(D)**, aged **(E)** or aged P7C3 **(F)** IVM oocytes. Scale bars: 30 μm **(G)** Quantification of NAD(P)H autofluorescence in from young, aged and aged oocytes exposed to two different concentrations of NAMPT stimulation by P7C3 during IVM. 10-20 oocytes isolated from 3-6 animals were analyzed. The experiment was repeated three times. Statistical analysis by one-way ANOVA: p<0.001; followed by Tukey’s multiple comparisons test: *p<0.05; ***p<0.001. **(H)** Effect of aging and NAMPT stimulation by P7C3 on oocyte ability to reach the MII stage after IVM. IVM was performed in pools of at least 15 oocytes from 3-6 mice in each experimental group. In brackets numbers of pools: young (n=11); aged (n=12); aged 1 µM P7C3 (n=7); aged 5 µM P7C3 (n=7). Statistical analysis by one-way ANOVA: p=0.006; followed by Tukey’s multiple comparisons test: *p<0.05; ***p<0.001. Different letters indicate *p*<0.05. (I) Effect of aging and NAMPT stimulation by P7C3 on ATP production in IVM oocytes. Pools of 5-8 oocytes from 3-6 mice were measured. In brackets numbers of pools: young (n=5); aged (n=10); aged 1 µM P7C3 (n=4); aged 5 µM P7C3 (n=6). Statistical analysis by Kruskal-Wallis test: not significant.

Based on changes in expression of genes involved in NAD^+^ metabolism and, more specifically, the downregulation of NAMPT (i.e., the rate-liming enzyme of NAD^+^ production), aged GV oocytes were exposed to different concentration of the NAMPT activator P7C3 during IVM. Bioluminescent NAD quantification revealed that aged oocytes reaching the MII stage after IVM presented lower levels of NAD^+^ in comparison to young IVM cells (Fig. 6A). Although not significant, a trend indicating a reduction total NAD content was found in aged IVM oocytes in comparison to young IVM cells (Fig. 6C). No differences were found regarding NADH levels in young and aged oocytes (Fig. 3B). The addition of 1 μM P7C3 in the IVM medium did not influence the amount of NAD^+^, but it effectively increases the amount of NADH and total NAD content (Fig. 6A-C).

In addition, we also evaluated the effects of different P7C3 concentrations by imaging technique exploiting the autofluorescence of NADH and NADPH in accordance with previous works [8, 23] (Fig. 6D-F). As shown in Fig. 6G, aged IVM oocytes presented a reduction of NAD(P)H levels in comparison to young IVM group. Both concentrations of P7C3 were able to increase intracellular NAD(P)H in comparison to aged IVM cells. The presence of 1 μM P7C3 restored NAD(P)H to levels similar to young IVM oocytes, whereas the 5 μM P7C3 induced an increase of NAD(P)H that was higher than that observed in young IVM oocytes.

### Addition of P7C3 improves the quality of meiotic spindle of aged oocytes

Given the beneficial effects of both P7C3 concentrations on NAD availability, we focused on the effects of NAMPT stimulation by P7C3 to improve meiosis resumption and MII spindle assembly in aged oocytes. At first, we observed that the ability of aged oocytes to resume meiosis was similar to young oocytes (Fig. 6H). The presence of 1 μM P7C3 in the IVM medium did not induce improvements in maturation rate, whereas the incubation with 5 μM P7C3 reduced significantly the ability of aged oocytes to reach the MII stage (Fig. 6H).

Beneficial effects of P7C3 at both tested concentrations were observed in meiotic spindle assembly and chromosome configuration. In fact, as shown in Table 2, the addition of 1 μM P7C3 in IVM medium had a positive impact on MII apparatus with a significant increase in the percentage of oocytes showing a normal spindle and correct DNA distribution, reaching the proportion observed in young IVM oocytes. The presence of 5 μM P7C3 also reduced the number of oocytes with abnormal MII apparatus and increased the proportion of oocytes with normal spindle and DNA configuration with levels similar to those observed in young controls.

### Aging influences the ability of mouse oocytes to respond to mitochondrial inhibitors of OXPHOS and alters oocyte bioenergetic profile

**Figure 7.**
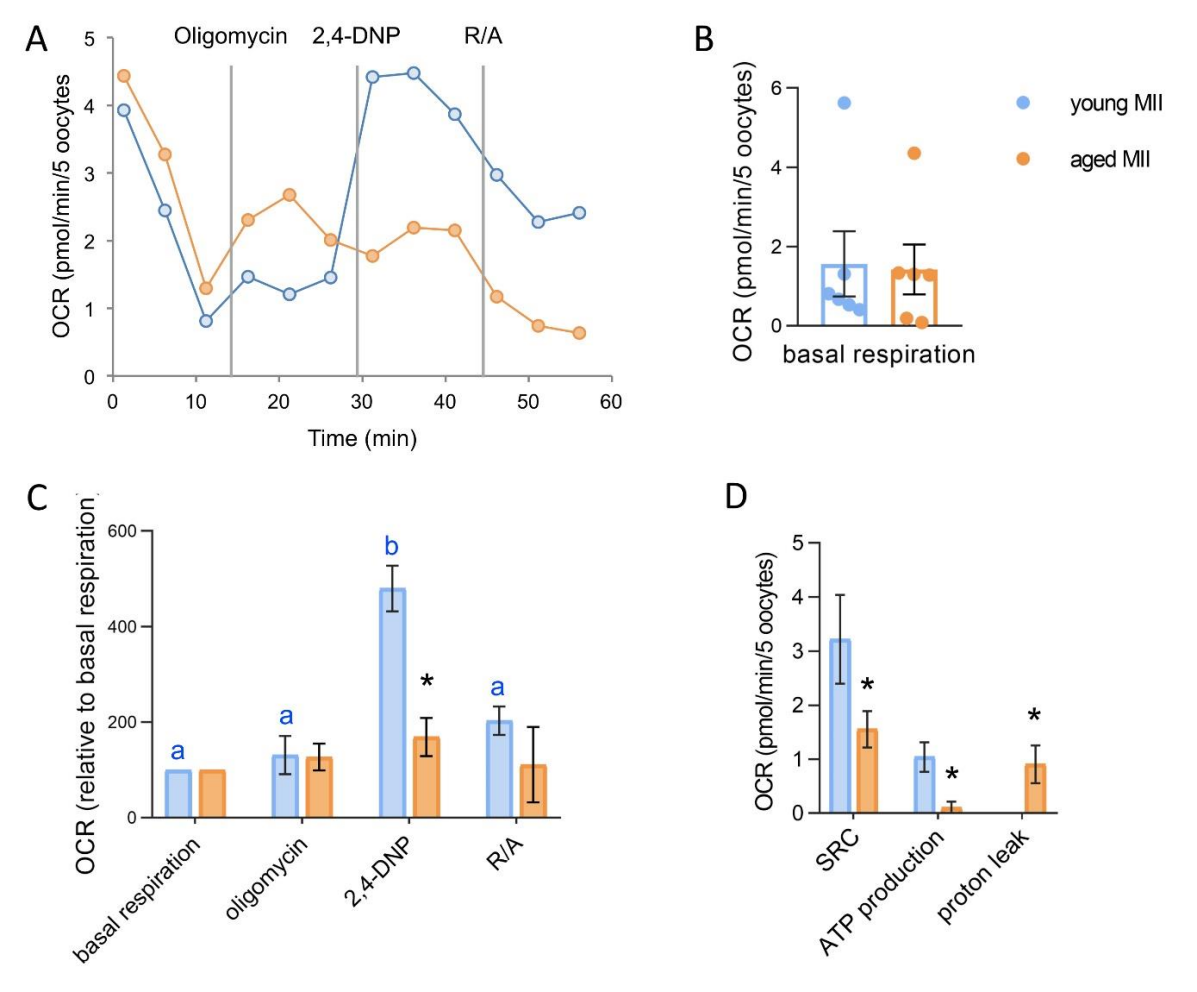
**Bioenergetic profile of young and aged MII oocytes. (A)** Representative profile of live measurements of changes OCR of young and aged MII oocytes upon injection of mitochondrial inhibitors. **(B)** Basal respiration (mean of third measure) of young and aged MII oocytes. Pools of 5-8 oocytes from 3-6 mice were measured. In brackets numbers of pools: young (n=6); aged (n=6). Statistical analysis by Mann Whitney test: not significant. **(C)** Mean values of OCR after oligomycin, 2,4-DNP and R/A of young and aged MII oocytes. Pools of 5-8 oocytes from 3-6 mice were measured. In brackets numbers of pools: young (n=6); aged (n=6). Oocyte response to addition of mitochondrial inhibitors was analyzed by one way ANOVA, followed by Student-Newman-Keuls multiple comparison. Different letters indicate a p<0.05 in young MII oocytes (blue). No differences were found in aged oocytes. *p<0.05 indicates differences in OCR between young and aged MII oocytes after unpaired t-test analysis. **(D)** Spare respiratory capacity (SRC), ATP production and proton leak obtained from live measurements of OCR. Statistical analysis by unpaired t-test *p<0.05 for SRC; or by Mann Whitney test *p<0.05 for ATP production and proton leak.

Live measurements of changes in oxygen consumption after addition of specific inhibitors of OXPHOS were used to study differences induced by the aging process in freshly ovulated MII oocytes (Fig. 7). By doing this we obtained the bienergetics profile and derivate information regarding the energetic status of the cells (i.e. SRC, ATP production, proton leak). As shown in Fig. 7A, upon loading on microplate for OCR analysis, both young and aged MII oocytes adjust their respiration, as demonstrated by the reduction of OCR from first to third measure. The comparison of the bioenergetic profile revealed that young and aged MII oocytes presented similar basal respiration and response to oligomycin (Fig. 7B, C). Interestingly, aged MII oocytes did not respond with a rise in OCR after the addition of the uncoupling agent 2,4-DNP, whereas young MII oocytes showed a marked boost in oxygen consumption after injection of 2,4-DNP. The final addition of R/A significantly reduced the OCR in young MII oocytes, while induced no changes in aged MII oocytes. In accordance with the lack of response to the uncoupler, we found that aged oocytes presented a reduced spare respiratory capacity, an indicator of reduced ability to cope with stressful conditions, and an increased proton leak (Fig. 7D). Finally, aged MII oocytes exhibited a significant reduction of ATP production, as shown in Fig. 7D.

**Table 2:** Distribution of oocytes with a normal, slightly aberrant, and aberrant MII spindle and chromosome configuration.

Experimental class	n.	normal spindle and chromosome distribution	slightly aberrant spindle and/or chromosome distribution	aberrant spindle and/or chromosome distribution	p value (to young)	p value (to aged)
**Young IVM**	38	20 (53%)	13 (34%)	5 (13%)	-	-
**Aged IVM**	28	6 (22%)	4 (14%)	18 (64%)	p=0.000093	-
**Aged IVM P7C3 1 µM**	25	15 (60%)	6 (24%)	4 (16%)	p=0.152439	p=0.0015
**Aged IVM P7C3 5 µM**	27	9 (33%)	10 (37%)	8 (30%)	p=0.174247	p=0.0301

Similarly to MII oocytes, young and aged ovarian oocytes at GV stage present a similar level of basal respiration (Supplementary Fig. 3A, B). Unlike MII oocytes, aged GV oocytes showed a significant increase in OCR upon addition of oligomycin, reaching levels higher than young GV oocytes (Supplementary Fig. 3C). The addition of the uncoupler 2,4-DNP induced the expected rise in OCR in both young and aged GV oocytes, an event which was not observed in aged ovulated MII oocytes. The final addition of R/A significantly reduced the OCR in both young and aged GV oocytes, but it was more pronounced in young cells. In contrast to MII oocytes, young and aged GV oocytes presented a similar spare respiratory capacity (Supplementary Fig. 3D). Finally, aged GV oocytes exhibited a significant reduction of ATP production in association with increased proton leak, as observed also in MII oocytes.

At the end of the measure, all oocytes analyzed under stereomicroscope were intact and showed no signs of degeneration.

### The presence of P7C3 in medium supports energetic demands of aged oocytes

**Figure 8.**
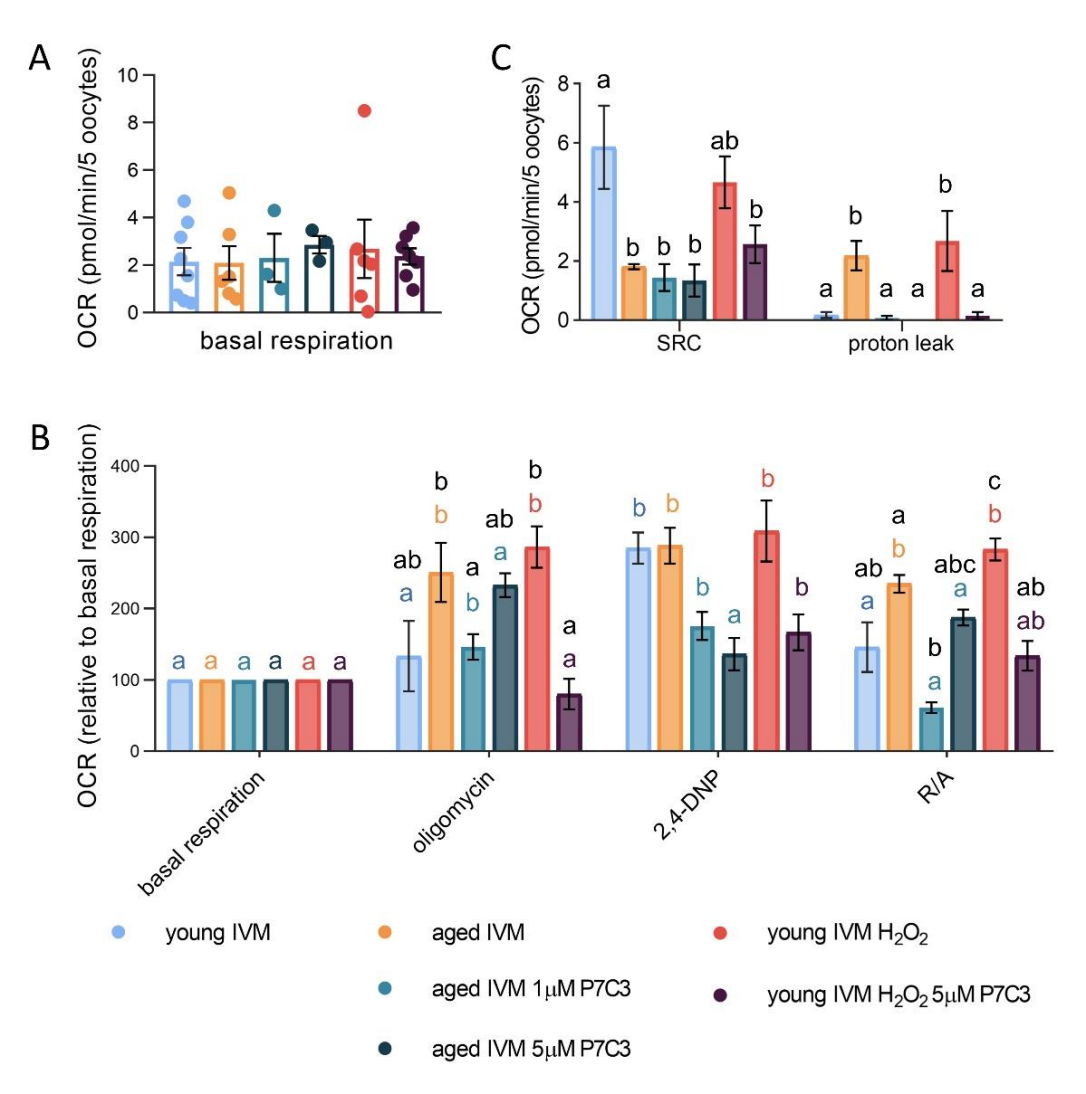
**Effect of NAMPT stimulation by P7C3 on bioenergetic profile of aged oocytes or young oocytes exposed to oxidative stress. (A)** Basal respiration (mean of third measure) of young IVM oocytes, aged IVM oocytes, aged oocytes exposed to 1 or 5 µM P7C3 during IVM, young oocytes stressed with H_2_O_2_ or exposed to P7C3 after stress with H_2_O_2_. Pools of 5-8 oocytes from 3-6 mice were measured. In brackets numbers of pools: young (n=8); aged (n=6); aged 1 µM P7C3 (n=3); aged 5 µM P7C3 (n=3); young H_2_O_2_ (n=6); young H_2_O_2_ 5 µM P7C3 (n=7). Statistical analysis by one-way ANOVA: not significant. **(B)** Bioenergetic profile of aged and young stressed oocytes and effect of NAMPT stimulation by P7C3. Mean values of OCR after oligomycin, 2,4-DNP and R/A of young IVM oocytes, aged IVM oocytes, aged oocytes exposed to 1 or 5 µM P7C3 during IVM, young oocytes stressed with H_2_O_2_ or exposed to P7C3 after stress with H_2_O_2_. Pools of 5-8 oocytes from 3-6 mice were measured. In brackets numbers of pools: young (n=8); aged (n=6); aged 1 µM P7C3 (n=3); aged 5 µM P7C3 (n=3); young H_2_O_2_ (n=6); young H_2_O_2_ 5 µM P7C3 (n=7). Oocyte response to addition of mitochondrial inhibitors was analyzed by one-way ANOVA, followed by Student-Newman-Keuls multiple comparison. Different letters indicate a p<0.05 in young (blue); aged (orange); aged 1 µM P7C3 (pale green); aged 5 µM P7C3 (green); young H_2_O_2_ (red); young H_2_O_2_ 5 µM P7C3 (purple) IVM oocytes. Differences in response to mitochondrial inhibitors among experimental groups were analyzed by one-way ANOVA, followed by Student-Newman-Keuls multiple comparison. Different letters (black) indicate a p <0.05. (C) Spare respiratory capacity (SRC) and proton leak obtained from live measurements of OCR. Statistical analysis by one-way ANOVA (p=0.0093) followed by Student-Newman-Keuls multiple comparison for SRC; or by Kruskal Wallis test (p=0.0075) followed by uncorrected Dunn’s test for multiple comparisons of proton leak. Different letters (black) indicate a p<0.05.

The potential beneficial role of NAD^+^ boosting on OXPHOS was analyzed after P7C3 supplementation in IVM medium. As observed for freshly ovulated MII oocytes, young and aged IVM oocytes did not present differences in basal respiration (Fig. 8A). In contrast to MII oocytes, upon IVM a significant increase in OCR upon oligomycin in the aged IVM oocytes is observed (Fig. 8B). The addition of P7C3 reduced this effect, with a significant reduction of OCR with the lowest P7C3 concentration (1 µM). Nevertheless, the lack of response of aged IVM oocytes to the uncoupler was not improved by the presence of P7C3. Unlike aged oocytes, the final addition of R/A in 1 µM P7C3 induced a significant reduction of respiration. This effect was not observed when oocytes were incubated in IVM medium supplemented with the higher concentration of P7C3 (i.e., 5 µM). Regarding additional information obtained from changes in OCR in response to inhibitors of mitochondrial OXPHOS elements, none of the P7C3 concentrations tested were able to ameliorate the spare respiratory capacity affected by aging (Fig. 8C). The presence of P7C3 had beneficial effects on reduction of proton leak, which was increased in aged oocytes (Fig. 8C). Unlike observed in freshly ovulated MII oocytes, ATP production did not change after IVM regardless of aging or the presence of P7C3 in IVM medium, as measured by luminescence quantification of ATP level (Fig. 6I).

### Oxidative stress recapitulates changes in bioenergetics profile of aged oocytes

**Figure 9.**
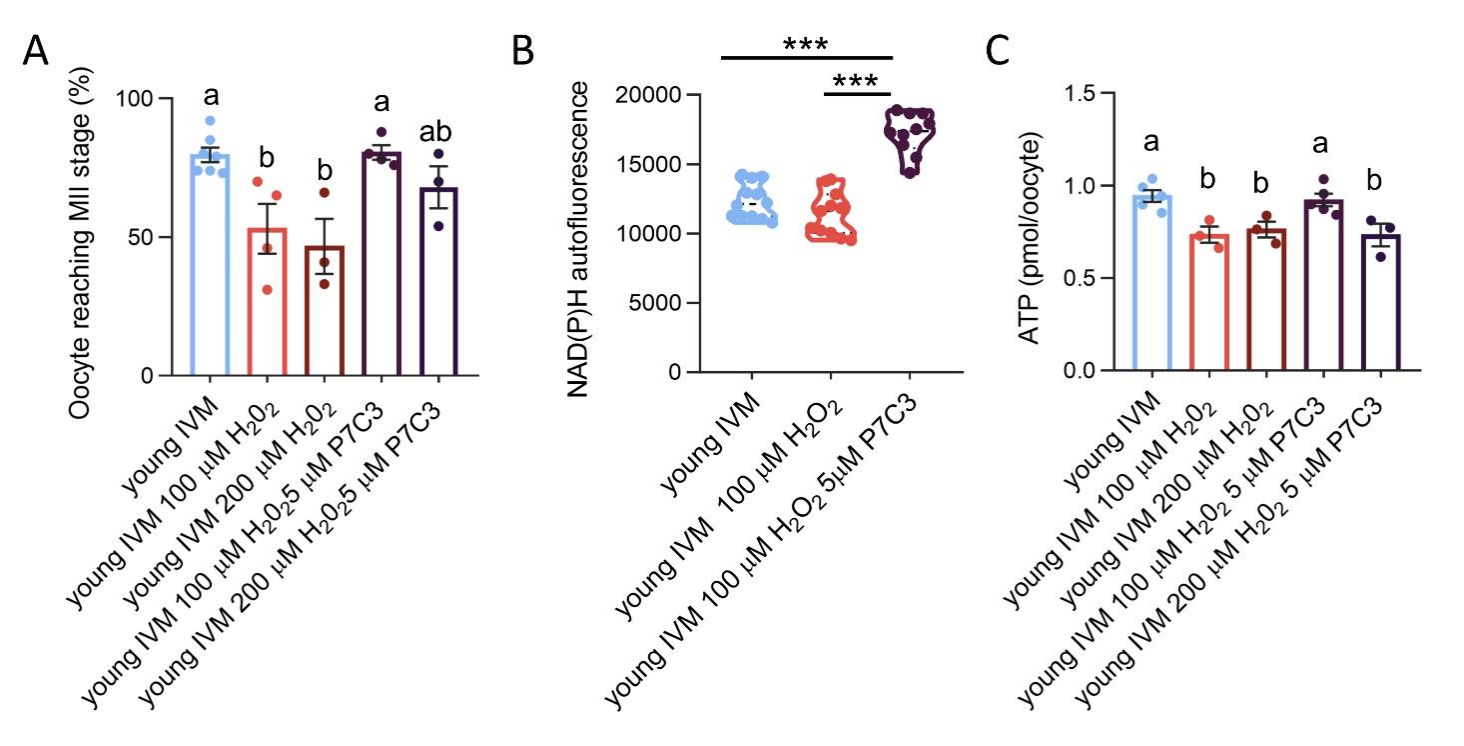
**Effect of NAMPT stimulation by P7C3 on IVM rate, NAD content and ATP production of young oocytes exposed to oxidative stress. (A)** Effect of exposure to oxidative stress and NAMPT stimulation by P7C3 on oocyte ability to reach the MII stage after IVM. IVM was performed in pools of at least 20 oocytes in each experimental group, isolated from 3-6 mice. In brackets numbers of pools: young (n=7); young 100 µM H_2_O_2_ (n=4); young 200 µM H_2_O_2_ (n=3); young 100 µM H_2_O_2_ 5 µM P7C3 (n=4); young 200 µM H_2_O_2_ 5 µM P7C3 (n=3). Statistical analysis by one-way ANOVA: p=0.0023 followed by Tukey’s multiple comparisons test. Different letters indicate p<0.05. **(B)** Quantification of NAD(P)H autofluorescence in from young, exposed to oxidative stress prior to IVM and matured in the presence or absence of NAMPT stimulation by P7C3. 10-20 oocytes isolated from 3-6 animals were analyzed. The experiment was repeated three times. Statistical analysis by one-way ANOVA: p<0.001; followed by Tukey’s multiple comparisons test: ***p<0.001. **(C)** Effect of exposure to oxidative stress and NAMPT stimulation by P7C3 on ATP production in IVM oocytes. Pools of 5 oocytes were measured. In brackets numbers of pools: young (n=5); young 100 µM H_2_O_2_ (n=3); young 200 µM H_2_O_2_ (n=3); young 100 µM H_2_O_2_ 5 µM P7C3 (n=5); young 200 µM H_2_O_2_ 5 µM P7C3 (n=3). Statistical analysis by one-way ANOVA p=0.003, followed by Tukey’s multiple comparisons test. Different letters indicate p<0.05.

The exposure of young oocytes to oxidative stress prior to IVM has a negative influence on the completion of meiosis at both H_2_O_2_ concentrations, with no differences between the two concentrations used (Fig. 9A). The analysis of bioenergetic profile of oocytes exposed to oxidative stress (minimum effective concentration of hydrogen peroxide) prior to IVM revealed profound changes in the oxygen consumption upon inhibition of mitochondrial OXPHOS elements (Fig. 8B). When focusing on basal respiration, we observed that stressed oocytes exhibited a level of OCR similar to young oocytes (Fig. 8A). Nevertheless, a significant increase in OCR was observed after oligomycin injection in oocytes exposed to OS prior to IVM, resembling what was observed in aged oocytes (Fig. 8B). OCR level after oligomycin in stress oocytes reached levels similar to OCR observed in young oocytes after depolarization by 2,4-DNP (Fig. 8B). The addition of 2,4-DNP in stressed oocytes was not able to induce any further increase in OCR (Fig. 8B). This lack of response was described also in aged oocytes, although stressed oocytes exhibited a higher OCR in comparison to aged cells (Fig. 8B). The final addition of R/A was not able to shut down oxygen consumption in stressed oocytes, with OCR remaining high and similar to that observed after oligomycin and 2,4-DNP (Fig. 8B).

As shown in Fig. 8C, stressed oocytes presented a spare respiratory capacity similar to that detected in young controls and higher than that observed in aged oocytes. Nevertheless, the calculation of this parameter did not consider that the stressed oocyte had a burst in OCR upon oligomycin, which is maintained but not increased after 2,4-DNP. Regarding proton leak, the exposure to oxidative stress induced an increase in comparison to young control oocytes (Fig. 8C). Finally, oocytes exposed to H_2_O_2_ prior to IVM presented a significant decrease of ATP production (Fig. 9C).

### Changes in bioenergetics induced by oxidative stress are prevented by the addition of P7C3 in IVM medium

Supplementation of IVM medium with P7C3 was able to increase NAD content and preserve the ability of young oocytes exposed to H_2_O_2_ to complete meiosis at both concentrations tested (Fig. 9A, B). Moreover, when oocytes were exposed for 10 min to 100 µM H_2_O_2_ prior to IVM, P7C3 had beneficial effects also on oocyte energy production, evaluated as ATP content, which is improved in comparison to H_2_O_2_ and similar to young controls (Fig. 9C). Conversely, when oocytes were exposed to the 200 µM H_2_O_2_ for 15 min prior to IVM, P7C3 was not able to sustain oocyte energetic demand, and ATP level was not recovered (Fig. 9C). In terms of bioenergetic profile, the presence of P7C3 in the IVM medium was effective in preventing the burst in oxygen consumption induced by the addition of oligomycin in oocytes exposed to 100 µM H_2_O_2_ (Fig. 8B). The addition of the uncoupling compound 2,4-DNP induced an increase in OCR in comparison to respiration observed after oligomycin injection in oocytes matured with P7C3 (Fig. 8B). However, the peak of oxygen consumption reached by young oocytes exposed to P7C3 IVM after OS was lower than that observed in H_2_O_2_-stressed oocytes (Fig. 8B). As observed in young oocytes, the final addition of R/A was not able to shut down oxygen consumption in comparison to 2,4-DNP in the young stressed P7C3 group, although OCR was lower than that observed in the stressed condition (Fig. 8B).

**Figure 10.**
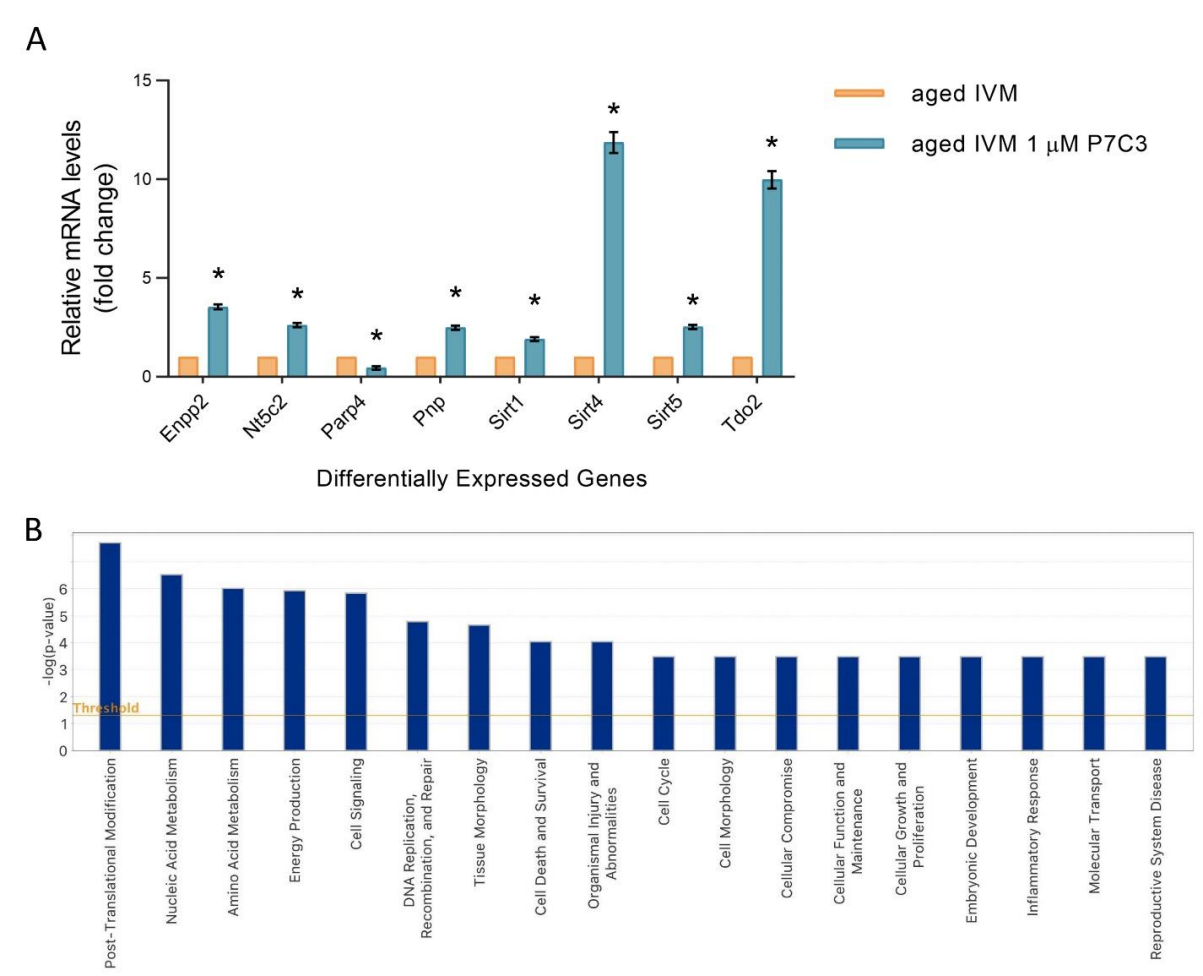
**Differentially expressed genes involved in NAD^+^ metabolism in aged oocytes exposed to NAMPT stimulation by P7C3 during IVM and Ingenuity pathway analysis (IPA)-generated functional analysis. (A)** Histograms of significant mean fold change values for all differentially expressed genes in aged oocytes exposed to P7C3 compared to controls. Pools of 25 oocytes isolated from 3-6 mice were employed. The experiment was repeated three times. Statistical analysis by paired t-test: ^*p^<0.05. **(B)** The bar-chart is generated based on a -log(p-value) threshold of 0.05 and indicates the main significant biological functions regulated by our gene dataset in aged oocytes exposed to P7C3.

Although the spare respiratory capacity of oocytes matured in the presence of P7C3 after stress was similar to the H_2_O_2_ oocytes, the value was lower than the young control group (Fig. 8C). As observed with aged cells, the addition of P7C3 was able to prevent the rise in proton leak presented by oocytes exposed to OS prior to IVM (Fig. 8C). Quantification of ATP content by luminescent technology revealed higher levels in stressed oocytes matured in the presence of P7C3 in comparison to oocytes matured in plain medium after the incubation with 100 μM H_2_O_2_. Conversely, P7C3 was not able to revert the effect of 200 μM H_2_O_2_ in terms of energy production (Fig. 9C).

### The addition of P7C3 to IVM medium ameliorates NAD^+^ metabolism of aged oocytes: IPA-inferred functional and network analysis of differentially expressed genes involved in NAD metabolism

Following statistical analysis of the expression of genes related to NAD^+^ biosynthetic and consuming pathways, a significant up-regulation of 7 genes (Enpp2, Nt5c2, Pnp, Sirti1, Sirt4, Sirt5 and Tdo2) was evidenced in aged oocytes which underwent IVM in the presence of NAD^+^ booster P7C3. On the other hand, one gene was found to be down-regulated, Parp4, in aged oocytes matured in the presence of P7C3 in comparison to aged oocytes (Fig. 10A).

**Figure 11.**
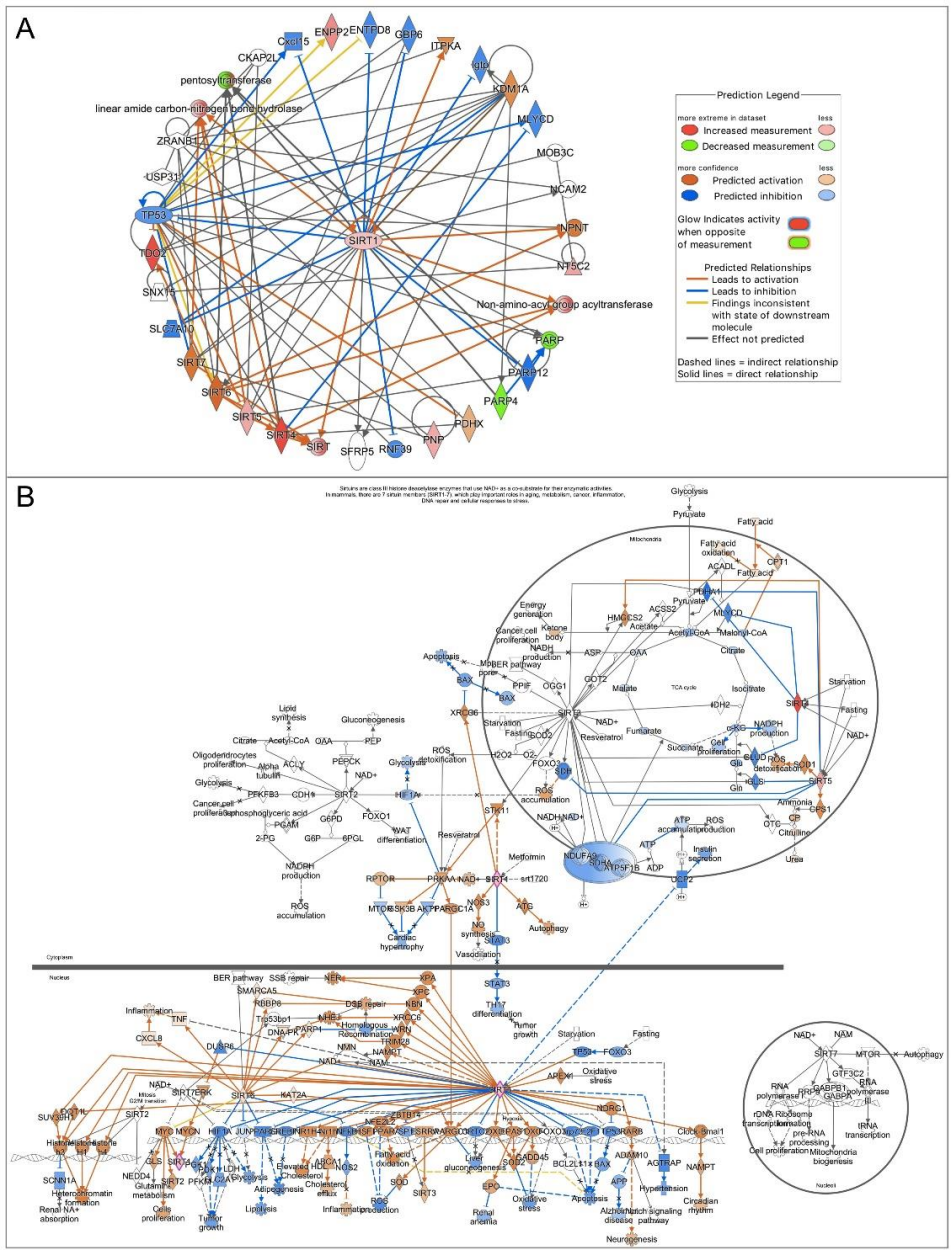
**Sirtuins are the central node of IPA-interfered target gene network. (A)** IPA-interfered target gene network for NAD^+^ metabolism of aged oocytes exposed to 1 µM P7C3 during IVM revealed that Sirt1 gene is the central node of IPA-interfered target gene network. In red the up-regulated genes, while in green the down-regulated ones. Blue arrow lines indicate a predicted inhibition, while orange arrow lines a predicted activation. **(B)** IPA-generated Sirtuin 1 Signaling Pathway: in this panel the main responses mediated by sirtuins under the action of P7C3 are depicted. In the nucleus there is a reduction of oxidative stress, apoptosis, improvement of DNA repair, epigenetic regulation through heterochromatin formation. In the mitochondria it is predicted increased ROS detoxification and mild reduction of ATP reduced ROS accumulation.

**Figure 12.**
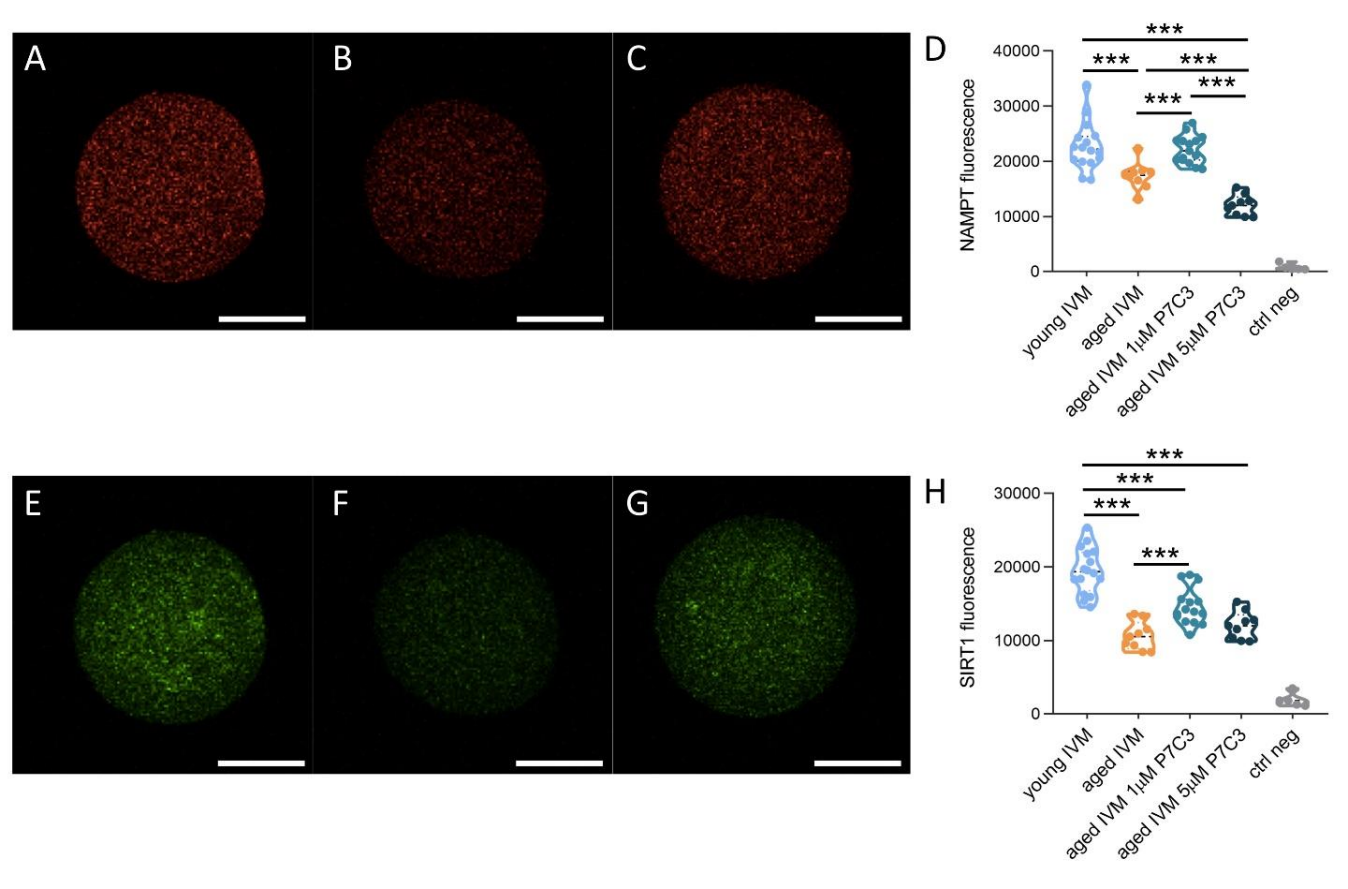
**Effect of aging and NAMPT stimulation by P7C3 on the protein level of NAMPT and SIRT1, key NAD^+^ producing and consuming enzymes**. Representative confocal images of NAMPT in young **(A)**, aged **(B)** or aged P7C3 **(C)** IVM oocytes. Scale bars: 30 μm. **(D)** Quantification of fluorescence intensity of NAMPT in young, aged or aged P7C3 IVM oocytes. 10-20 oocytes isolated from 3-6 animals were analyzed. The experiment was repeated three times. Statistical analysis by one-way ANOVA: p<0.001; followed by Tukey’s multiple comparisons test: ***p<0.001. Representative confocal images of SIRT1 in young **(E)**, aged **(F)** or aged P7C3 **(G)** IVM oocytes. Scale bars: 30 μm. **(H)** Quantification of fluorescence intensity of SIRT1 in young, aged or aged P7C3 IVM oocytes. 10-20 oocytes isolated from 3-6 animals were analyzed. The experiment was repeated three times. Statistical analysis by one-way ANOVA: p<0.001; followed by Tukey’s multiple comparisons test: ***p<0.001.

IPA functional, network and pathway analyses were carried out as previously described for the physiological aged murine oocytes. The 8 differentially expressed genes in the aged oocytes following the addition of P7C3 in the IVM medium, were found to be involved in 19 main functions, with the most relevant ones being the following: post-translational modifications, nucleic acid metabolism, amino acid metabolism, energy production, lipid metabolism and cell signaling (Fig. 10B, Supplementary Fig. 4).

Molecular network generated after IPA analysis of genes differentially expressed in the P7C3 group revealed that SIRTs represent the core of the diagram (Fig. 11A, Supplementary Fig. 5). The top IPA-derived network generated after the comparison of aged oocytes exposed to P7C3 in comparison to aged oocytes with a score equal to 25 (data not shown) is centered around the key node gene Sirt1 (Fig. 11A). In the nucleus, increased level of SIRT1 from dataset leads to the predicted activation of mitochondrial unfolded protein response, oxidative metabolism, mitochondrial biogenesis, also connected with the predicted increased of NAMPT and NAD^+^ bioavailability. As shown in Fig. 11B, the main responses mediated by SIRTs under the action of P7C3 are the reduction of oxidative stress, apoptosis, improvement of DNA repair, epigenetic regulation through heterochromatin formation at nuclear level. In the mitochondria increased ROS detoxification and reduced ROS accumulation, together with a mild reduction of ATP, are predicted.

### Low concentration of P7C3 in IVM medium increased the protein level of NAMPT and SIRT1, key enzymes in NAD^+^ production and utilization, altered in the aged oocytes

Since NAD^+^ production is influenced by NAMPT and controls the activity of sirtuins, we evaluated the amount of NAMPT and SIRT1 in aged oocytes exposed to different P7C3 concentrations during IVM. Our results provide evidence for a reduction of NAMPT protein level in aged oocytes in comparison to young cells. The presence of 1 μM P7C3 increases the amount of NAMPT affected by the aging process, reaching levels similar to young IVM oocytes. Interestingly, 5 μM P7C3 induced a further reduction of NAMPT levels in comparison to aged IVM oocytes (Fig. 12A-D).

Finally, our results confirmed that SIRT1 protein level was decreased in aged oocytes reaching the MII stage after IVM in comparison to young IVM oocytes. The presence of 1 μM P7C3 increases SIRT1 protein levels in the aged oocyte. By contrast, the addition of 5 μM P7C3 in IVM medium did not induce any change in SIRT1 levels in aged oocytes (Fig. 12E-H).

### P7C3 does not counteract aging like changes induce by oxidative stress on the protein expression of key enzymes in NAD^+^ metabolism

Similarly to the aging process, the exposure of young oocytes to oxidative stress prior to IVM altered the expression of NAMPT and SIRT1, key NAD^+^ producing and consuming enzymes respectively (Supplementary Fig. 6A). Interestingly, the presence of P7C3 in IVM medium induced a further reduction of NAMPT protein level in young, stressed oocytes. Regarding SIRT1 protein level, no differences were observed in oocytes exposed to H_2_O_2_ in the presence or absence of P7C3 (Supplementary Fig. 6B).

## DISCUSSION

**Figure 13.**
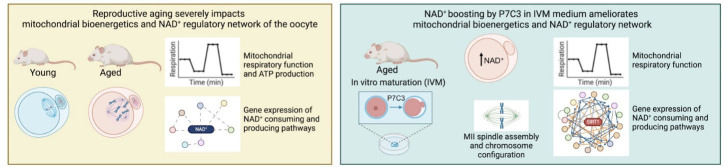
Graphical abstract reporting experimental design and main conclusions.

Recent literature evidence that NAD^+^ metabolism is relevant for the maintenance of fertility potential [16]. Numerous observations have suggested that increasing NAD^+^ availability may represent a possible strategy to improve the quality of oocytes isolated from reproductively aged mice and women [8, 19, 25]. In this context, the present study revealed the detrimental effects of aging on the expression of genes participating in NAD^+^ biosynthetic and NAD^+^ consuming pathways in MII oocytes. Moreover, in this study NAMPT stimulation during IVM by using P7C3 was demonstrated to ameliorate oocyte competence and mitochondrial activity in association with the modulation of genes involved in NAD^+^ metabolism (Fig. 13).

### Aging modulated NAD^+^ biosynthetic and NAD^+^ consuming pathways in mouse oocytes

An overall significant age-related modulation of genes related to the metabolism of NAD^+^ was found in mouse oocytes. Pathway and network analysis revealed a down-regulation of NAD^+^ biosynthesis due to a reduction of both NAD^+^ *de novo* production and NAD^+^ salvage (Fig. 1). Regarding the kynurenine pathway, we observed a reduction of Qprt, the enzyme that converts QA to NAMN and commits the pathway to NAD^+^ biosynthesis. This supports previous findings that showed that Qprt deletion accelerates ovarian aging [13]. Regarding the Preiss-Handler pathway, decreased levels of Nadsyn1 were detected in aged oocytes, as compared to young controls, thus suggesting that a reduction of NAD^+^ production from NaAD, the metabolite obtained from NAMN by NMNATs (Fig. 1). Finally, the observation of a reduced abundance of transcripts for Nampt may reveal a down-regulation of the NAD^+^ salvage pathway, in which NAMPT serves as the rate-limiting enzyme [4, 5]. Altered NAD^+^ production in the aged oocytes may influence cell homeostasis by reducing the activity of a plethora of NAD^+^-dependent enzymes. This condition could be further aggravated by the reduced transcriptional expression of NAD^+^ consuming enzymes e.g. Cd38, members of PARP (Tnks, Tnks2) and SIRT (Sirt3, Sirt4) families. Accordingly, IPA pathway and network analysis predicted a reduction of oocyte metabolic processes, such as fatty acid and glucose metabolism, but also a lowered DNA repair and oxidative metabolism, including ROS detoxification, which may lead to oxidative cell damage and to the activation of mitochondrial UPR.

Overall, these observations represent an important contribution to the understanding the reduction of NAD^+^ observed in the aged oocyte providing details of the possible molecular mechanisms underlying the beneficial effects exerted by NAD^+^ boosting therapies [8, 9, 12, 13] (Fig. 1). Our analysis can help to explain the efficacy of NAD^+^ precursors supplementation as oocyte anti-aging intervention suggested by others [8, 9, 12, 13]. The efficacy of NMN might be related to its reduced abundance as a consequence of reduced expression of NAMPT, that we have described in aged oocytes. NR supplementation can also contribute to increase NMN pool, through the activity NRKs, whose expression does not change upon aging in the oocyte [12]. NAD^+^ obtained from NMN through the activity of NMNATs can be endangered by the age-related reduced expression of Nmnat2 [12], the most expressed isoform in the oocyte [26]. Regarding the beneficial effects of NA supplementation in IVM medium of aged oocytes [12], this occurs although the NAD^+^ biosynthetic pathways are compromised. Indeed, NA utilization requires the activity of NMNATs and NADSYN, whose transcriptional expression is reduced with aging, revealing the possible presence of compensatory and/or regulatory mechanisms.

Our study reports a reduction of Nampt transcript in aged oocytes, in association with reduced NAMPT protein level. Nevertheless, other researchers found out no changes or increased Nampt expression with aging [12, 27]. This discrepancy is likely related to the fact that, in contrast to previous studies reporting data on GV oocytes, our analysis has been performed on oocytes at MII stage. This may suggest that the aging process does not negatively interferes with the accumulation of Nampt transcripts during oogenesis for adequate NAD^+^ production and that reduced Nampt expression in the aged MII oocyte is related to intense utilization of this transcript during meiotic maturation or to its reduced stability or to degradation.

### Stimulation of NAMPT ameliorated the competence of the aged oocyte

Overall, these observations support our working hypothesis on the effect of exposure of aged oocytes to P7C3 during IVM. This approach has been previously employed by Zhuan *et al*. [27], who revealed P7C3 efficacy on ATP levels. Our data allowed us to discover that the use of a NAMPT activator during IVM effectively increased NAD content in the aged oocyte. Consistently, we proved that the addition of P7C3 during IVM improved the competence of oocytes that reach the MII stage, as evidenced by reduction of spindle and chromosome alteration rates, reaching levels similar to those detected in young controls. Here, we observed that the aged oocyte is characterized by a reduced NAMPT protein expression and that the presence of P7C3 not only stimulates NAMPT activity but also promotes its protein expression. The key role of NAMPT in the first meiotic division has been previously described [17]. In particular, NAMPT has been involved in the regulation of asymmetric division and spindle size, probably through the Mos/MAPK-dependent pathway [27]. Moreover, NAMPT co-localizes with the mitochondrial pool surrounding the spindle [17] suggesting the strict link between this enzyme and mitochondrial functioning. Beneficial effects of NAMPT stimulation may be linked to tubulin acetylation operated by SIRT2, that is considered a spindle modulator [17]. Similarly to aging, NAMPT has found to be reduced in oocytes from obese mice [28]. Overexpression of NAMPT in these oocytes has been demonstrated to reduce ROS production and meiotic defects, thus proposing NAMPT as a key element for the maintenance of redox balance and normal meiosis [28].

Surprisingly, despite the positive effects of P7C3 on the oocyte aging phenotype, we found that the P7C3 when used at 5 µM reduced the number of aged oocytes reaching the MII stage after IVM. This reveals an upper limit to P7C3 tolerability or the increased formation of NMN, the product of NAMPT reaction. This could lead to an excess of NMN degradation to NAM, a sirtuin and PARP inhibitor with potential deleterious effects [8, 29].

### Stimulation of NAMPT ameliorated mitochondrial bioenergetics of the aged oocyte

Before focusing on the effects of NAMPT stimulation on mitochondrial bioenergetics, we characterized the bioenergetic profile of oocytes ovulated by aged mice. Based on the measurement of the real-time oxygen consumption rate, we found that aged MII oocytes exhibited low responsiveness to mitochondrial inhibitors in comparison to young oocytes, as evidence of low OXPHOS activity. Indeed, a reduction of ATP production was observed in aged oocytes in accordance with literature [30-32]. These observations may be related to altered integrity of inner mitochondrial membrane (IMM) and increased IMM permeability related to activation of the mitochondrial permeability transition pores (mtPTPs). In aging cells, this causes mitochondria depolarization, thus impairing OXPHOS, releasing Ca^2+^ and mitochondrial ROS and depleting cellular NAD^+^ [33]. Similar results on age-dependent reduction of sensitivity to mitochondrial inhibitors have been obtained in stromal ovarian cells isolated from aged mice [34], thus suggesting that this phenomenon involves both the germinal and somatic ovarian compartments. On the other hand, reduced OXPHOS activity could be linked to age-related down-regulation of both mitochondrial and nuclear encoded proteins functioning in the ETC [32, 35, 36]. Moreover, aged oocytes exhibited a reduced spare respiratory capacity, and this could reflect an increased sensitivity to sudden surges of ATP demand, as well as to reduced ability to increase cellular metabolic activity upon stressing conditions, as described in aged somatic post-mitotic cells [37]. Finally, we have found an increase in proton leak in aged oocytes, which has been reported in aging using multiple tissues and cell types [38]. When mediated by uncoupling proteins such as ANT1, proton leak represents a means by which cells reduce OXPHOS activity to limit mitochondrial ROS production [38]. On the other hand, an excessive proton leak increases the mitochondrial workload, resulting in a decline in respiratory efficiency, decreasing ATP output, and exacerbating electron leak and superoxide anion generation [38].

When aged oocytes were matured *in vitro* in the presence of P7C3 an improvement in OXPHOS activity was observed. This was evidenced by increased responsiveness to rotenone and antimycin A, the inhibitors of ETC complexes I and III, respectively. This may be due to P7C3-dependent increased availability of NAD^+^, as the redox carrier receiving hydride from TCA cycle and FAO to be donated to complex I for ATP synthesis through mitochondrial OXPHOS [5, 39]. In addition, P7C3-treated oocytes exhibited a reduced proton leak, an effect that could contribute to improve the efficacy of mitochondrial activity. This effect was not observed with the highest concentration of P7C3 (i.e., 5 µM), further supporting the notion that proper P7C3 dosing is required to obtain beneficial effects [8], also taking into account that in some cellular models P7C3 was found to trigger the activation of specific signaling pathways (e.g., PKA/Akt) [38].

Unfortunately, mitochondrial recovery from aging under P7C3 stimulation seemed to be partial. In fact, the positive P7C3-induced effects on OXPHOS were not paralleled by an increased ATP production, and were not associated with improved spare respiratory capacity, thus suggesting that ETC function may be irreversibly compromised by aging.

### NAMPT stimulation during IVM improved competence and mitochondrial bioenergetics under oxidative stress

To investigate whether the oxidative stress experienced by the aged oocyte may contribute to age-related mitochondrial bioenergetics, we exposed young oocytes to hydrogen peroxide prior to IVM. Results from these experiments allowed us to assess differences and similarities between young stressed oocytes and aged oocytes. Unlike aged oocytes, young stressed cells did not have a reduction of NAD content, but presented a similar reduction in protein levels of NAMPT and SIRT1, the key enzyme orchestrating oocyte adaptive response to oxidative stress [40-42].

A relevant finding is that, although reduced NAMPT expression, the presence of P7C3 during IVM increased NAD content and improved the ability of young oocytes to respond to H_2_O_2_ in terms of completion of meiosis and energy production, evaluated as ATP content. However, when exposed to mitochondrial inhibitors, stressed oocytes had a peculiar behavior, which resembled that shown by aged oocytes. The significant new result described herein is the observation of an oligomycin-induced proton uncoupling that is evidenced by a burst of OCR. This uncharacteristic response to oligomycin has been previously reported by Hearne *et al.* [43] in hepatocellular carcinoma cell line when mitochondrial substrates pyruvate and lactate were employed as energetic source [44]. Of note, these are the only energy substrates utilized by the oocyte. In contrast to observations by Hearne *et al.* [43], the stressed oocyte was not able to reverse the oligomycin-induced uncoupling, even when complex I and III inhibitors were added. Interestingly, when oocytes were matured in the presence of P7C3, the addition of oligomycin did not induce the uncoupling response, and the addition of the subsequent inhibitors induced the expected changes in OCR. Together with the prevention of massive proton leak, these observations reveal that promotion of NAD^+^ metabolism by P7C3 was able to restore mitochondrial functionality in oocytes exposed to oxidative stress. Therefore, present results support the hypothesis that peculiar responses to oligomycin may reveal additional information regarding mitochondrial response to stress.

### NAMPT stimulation during IVM influenced the expression of genes involved in NAD^+^ metabolism in the aged oocyte

The observed improvements of P7C3 in aged oocytes are associated to changes in the expression of genes responsible for NAD^+^ biosynthesis and consumption. Among the up-regulated genes, we found Tdo2, Enpp2, Pnp, and Nt5c2. The likely increase in the availability of Tdo2, an enzyme for tryptophan utilization in the kynurenine pathway, suggests a promotion of NAD^+^ biosynthesis in P7C3 oocytes. This result may reveal an interesting interplay among different pathways responsible for NAD^+^ generation. Enpp2, also known as autotaxin, is a NAD^+^ binding protein that modulates NAD^+^ levels directly through its enzymatic activity and indirectly by the generation of lysophosphatidic acid (LPA), a bioactive lipid molecule [45, 46]. LPA activates the G protein-coupled receptor LPAR1, which induces the expression of key NAD^+^ biosynthetic enzyme [45]. Moreover, ENPP2-derived LPA can inhibit CD38, thereby repressing NAD^+^ consumption and maintaining its availability for cellular processes [46]. Pnp and Nt5c2 are two other NAD^+^ binding proteins and participate in the NAD^+^ synthesis [47].

Regarding genes coding for NAD^+^ consuming enzymes, transcripts of three sirtuins (SIRTs) were found to be increased in aged oocytes exposed to P7C3, whereas Parp4 was the only transcript found to be downregulated. Of note, sirtuin activation represents the core of IPA pathway and network analysis, with mRNAs of Sirt1, Sirt4 and Sirt5 detected as up-regulated genes in aged P7C3-treated oocytes, and Sirt6 predicted to increase. Among genes predicted to increase are Sirt2 and Sirt3, which were not found up-regulated in our analysis. This could be due to limits of the technique in combination with the scarcity of the sample, which may hide possible small differences. Protein analysis confirmed that NAMPT stimulation by P7C3 increased SIRT1 level, counteracting its age-related decline. In the oocytes SIRTs have an important role in the acquisition of competence being associated with oxidative stress defense, adaptive response to nutrient status and meiosis [10, 40, 41, 48]. Down-regulation of Parp4, a NAD^+^ consuming enzyme involved in DNA repair, suggests a condition of reduced DNA damage in aged P7C3 oocytes [10].

## Conclusions

Our study revealed that NAD^+^ level in the aged oocyte could be impaired as a consequence of the altered expression of genes involved in all of the three major NAD^+^ biosynthetic pathways. Reduced expression of relevant NAD^+^ consuming enzymes may contribute further to the deregulation of NAD^+^ metabolism that impacts the competence of aged oocytes. By real-time measurements of oxygen consumption, we demonstrate for the first time that aging profoundly affects oocyte mitochondrial bioenergetics. Finally, we have demonstrated that NAMPT stimulation by P7C3 during IVM has the potential to improve the competence of the aged oocyte by acting on mitochondrial activity and by modulating genes involved in NAD^+^ metabolism. Unfortunately, although P7C3 has the ability to counteract the effects of oxidative stress, this compound exerts partial effects on aged oocytes. Moreover, the finding of some detrimental effects of high P7C3 dose claims the need for the definition of P7C3 tolerability in aged oocytes. Indeed, P7C3 is known to trigger the activation of pathways not related to NAD^+^ production [38] that may contribute to the observed negative effects. Full definition of such aspects would be relevant to establish whether supplementing medium of advanced maternal-age oocytes with this compound could be a treatment option worth exploring. Overall, our study contributes to expanding the knowledge required to establish effective and safe NAD^+^ boosting interventions to alleviate the effects of advanced maternal age on fertility and to explore their potential in redox-related fertility disorders.

## Supplementary Materials

The Supplementary data can be found online at: www.aginganddisease.org/EN/10.14336/AD.2024.0241.

## References

[1] Xiao W, Wang R-S, Handy DE, Loscalzo J (2018). NAD(H) and NADP(H) Redox Couples and Cellular Energy Metabolism. Antioxid Redox Signal, 28:251-272. 28648096 10.1089/ars.2017.7216 PMC5737637

[2] Kulikova VA, Gromyko DV, Nikiforov AA (2018). The Regulatory Role of NAD in Human and Animal Cells. Biochem Mosc, 83:800-812. 10.1134/S0006297918070040 30200865

[3] Olgun A (2009). Converting NADH to NAD+ by nicotinamide nucleotide transhydrogenase as a novel strategy against mitochondrial pathologies during aging. Biogerontology, 10:531-534. 18932012 10.1007/s10522-008-9190-2

[4] Yang Y, Sauve AA (2016). NAD+ metabolism: Bioenergetics, signaling and manipulation for therapy. Biochim Biophys Acta, 1864:1787-1800. 27374990 10.1016/j.bbapap.2016.06.014 PMC5521000

[5] Xie N, Zhang L, Gao W, Huang C, Huber PE, Zhou X, et al. (2020). NAD+ metabolism: pathophysiologic mechanisms and therapeutic potential. Signal Transduct Target Ther, 5:227. 33028824 10.1038/s41392-020-00311-7 PMC7539288

[6] Chu X, Raju RP (2022). Regulation of NAD+ metabolism in aging and disease. Metabolism, 126:154923. 34743990 10.1016/j.metabol.2021.154923 PMC8649045

[7] Shi H, Enriquez A, Rapadas M, Martin EMMA, Wang R, Moreau, et al. (2017). NAD Deficiency, Congenital Malformations, and Niacin Supplementation. N Engl J Med, 377:544-552. 28792876 10.1056/NEJMoa1616361

[8] Bertoldo MJ, Listijono DR, Ho W-HJ, Riepsamen AH, Goss DM, Richani D, et al. (2020). NAD+ Repletion Rescues Female Fertility during Reproductive Aging. Cell Rep, 30:1670-1681.e7. 32049001 10.1016/j.celrep.2020.01.058 PMC7063679

[9] Miao Y, Cui Z, Gao Q, Rui R, Xiong B (2020). Nicotinamide Mononucleotide Supplementation Reverses the Declining Quality of Maternally Aged Oocytes. Cell Rep, 32:107987. 32755581 10.1016/j.celrep.2020.107987

[10] Pollard C-L, Gibb Z, Hawdon A, Swegen A, Grupen CG (2021). Supplementing media with NAD+ precursors enhances the in vitro maturation of porcine oocytes. J Reprod Dev, 67:319-326. 34408103 10.1262/jrd.2021-080 PMC8568614

[11] Wang S, Sun M, Yu L, Wang Y, Yao Y, Wang D (2018). Niacin Inhibits Apoptosis and Rescues Premature Ovarian Failure. Cell Physiol Biochem Int J Exp Cell Physiol Biochem Pharmacol, 50:2060-2070. 10.1159/000495051 30415247

[12] Wu X, Hu F, Zeng J, Han L, Qiu D, Wang H, et al. (2019). NMNAT2-mediated NAD+ generation is essential for quality control of aged oocytes. Aging Cell, 18:e12955. 30909324 10.1111/acel.12955 PMC6516161

[13] Yang Q, Li H, Wang H, Chen W, Zeng X, Luo X, et al. (2023). Deletion of enzymes for de novo NAD+ biosynthesis accelerated ovarian aging. Aging Cell, 22:e13904. 37332134 10.1111/acel.13904 PMC10497836

[14] Tatone C, Amicarelli F, Carbone MC, Monteleone P, Caserta D, Marci R, et al. (2008). Cellular and molecular aspects of ovarian follicle ageing. Hum Reprod Update, 14:131-142. 18239135 10.1093/humupd/dmm048

[15] Shen L, Liu J, Luo A, Wang S (2023). The stromal microenvironment and ovarian aging: mechanisms and therapeutic opportunities. J Ovarian Res, 16:237. 38093329 10.1186/s13048-023-01300-4 PMC10717903

[16] Liang J, Huang F, Song Z, Tang R, Zhang P, Chen R (2023). Impact of NAD+ metabolism on ovarian aging. Immun Ageing, 20:70. 38041117 10.1186/s12979-023-00398-w PMC10693113

[17] Wei Z, Greaney J, Loh W-GN, Homer HA (2020). Nampt-mediated spindle sizing secures a post-anaphase increase in spindle speed required for extreme asymmetry. Nat Commun, 11:3393. 32636388 10.1038/s41467-020-17088-6 PMC7341875

[18] Yang Q, Chen W, Cong L, Wang M, Li H, Wang H, et al. (2024). NADase CD38 is a key determinant of ovarian aging. Nat Aging, 4:110-128. 38129670 10.1038/s43587-023-00532-9 PMC10798903

[19] Huang P, Zhou Y, Tang W, Ren C, Jiang A, Wang X, et al. (2022). Long-term treatment of Nicotinamide mononucleotide improved age-related diminished ovary reserve through enhancing the mitophagy level of granulosa cells in mice. J Nutr Biochem, 101:108911. 34801690 10.1016/j.jnutbio.2021.108911

[20] Konstantinidou F, Budani MC, Sarra A, Stuppia L, Tiboni GM, Gatta V (2021). Impact of Cigarette Smoking on the Expression of Oxidative Stress-Related Genes in Cumulus Cells Retrieved from Healthy Women Undergoing IVF. Int J Mol Sci, 22:13147. 34884952 10.3390/ijms222313147 PMC8658611

[21] Tatone C, Di Emidio G, Barbaro R, Vento M, Ciriminna R, Artini PG (2011). Effects of reproductive aging and postovulatory aging on the maintenance of biological competence after oocyte vitrification: insights from the mouse model. Theriogenology, 76:864-873. 21705053 10.1016/j.theriogenology.2011.04.017

[22] Placidi M, Vergara T, Casoli G, Flati I, Capece D, Artini PG, et al. (2023). Acyl-Carnitines Exert Positive Effects on Mitochondrial Activity under Oxidative Stress in Mouse Oocytes: A Potential Mechanism Underlying Carnitine Efficacy on PCOS. Biomedicines, 11:2474. 37760915 10.3390/biomedicines11092474 PMC10525604

[23] Dumollard R, Ward Z, Carroll J, Duchen MR (2007). Regulation of redox metabolism in the mouse oocyte and embryo. Development, 134:455-465. 17185319 10.1242/dev.02744

[24] Nohales-Córcoles M, Sevillano-Almerich G, Di Emidio G, Tatone C, Cobo AC, Dumollard R, et al. (2016). Impact of vitrification on the mitochondrial activity and redox homeostasis of human oocyte. Hum Reprod, 31:1850-1858. 27251202 10.1093/humrep/dew130

[25] Smits MAJ, Schomakers BV, van Weeghel M, Wever EJM, Wüst RCI, Dijk F, et al. (2023). Human ovarian aging is characterized by oxidative damage and mitochondrial dysfunction. Hum Reprod, 38:2208-2220. 37671592 10.1093/humrep/dead177 PMC10628503

[26] Berger F, Lau C, Dahlmann M, Ziegler M (2005). Subcellular compartmentation and differential catalytic properties of the three human nicotinamide mononucleotide adenylyltransferase isoforms. J Biol Chem, 280:36334-36341. 16118205 10.1074/jbc.M508660200

[27] Zhuan Q, Li J, Du X, Zhang L, Meng L, Cheng K, et al. (2022). Nampt affects mitochondrial function in aged oocytes by mediating the downstream effector FoxO3a. J Cell Physiol, 237:647-659. 34318928 10.1002/jcp.30532

[28] Wang H, Zhu S, Wu X, Liu Y, Ge J, Wang Q, et al. (2021). NAMPT reduction-induced NAD+ insufficiency contributes to the compromised oocyte quality from obese mice. Aging Cell, 20:e13496. 34662475 10.1111/acel.13496 PMC8590097

[29] Hwang ES, Song SB (2020). Possible Adverse Effects of High-Dose Nicotinamide: Mechanisms and Safety Assessment. Biomolecules, 10:687. 32365524 10.3390/biom10050687 PMC7277745

[30] Iwata H, Goto H, Tanaka H, Sakaguchi Y, Kimura K, Kuwayama T, et al. (2011). Effect of maternal age on mitochondrial DNA copy number, ATP content and IVF outcome of bovine oocytes. Reprod Fertil Dev, 23:424-432. 21426860 10.1071/RD10133

[31] Simsek-Duran F, Li F, Ford W, Swanson RJ Jr, HW J, Castora FJ (2013). Age-Associated Metabolic and Morphologic Changes in Mitochondria of Individual Mouse and Hamster Oocytes. PLOS ONE, 8:e64955. 23741435 10.1371/journal.pone.0064955 PMC3669215

[32] Kobayashi H, Yoshimoto C, Matsubara S, Shigetomi H, Imanaka S (2023). Altered Energy Metabolism, Mitochondrial Dysfunction, and Redox Imbalance Influencing Reproductive Performance in Granulosa Cells and Oocyte During Aging. Reprod Sci doi: 10.1007/s43032-023-01394-7. 37917297

[33] Rottenberg H (2023). The Reduction in the Mitochondrial Membrane Potential in Aging: The Role of the Mitochondrial Permeability Transition Pore. Int J Mol Sci, 24:12295. 37569671 10.3390/ijms241512295 PMC10418870

[34] Umehara T, Winstanley YE, Andreas E, Morimoto A, Williams EJ, Smith KM, et al. Female reproductive life span is extended by targeted removal of fibrotic collagen from the mouse ovary. Sci Adv, 8:eabn4564. 35714185 10.1126/sciadv.abn4564 PMC9205599

[35] Hamatani T, Carter MG, Sharov AA, Ko MSH (2004). Dynamics of global gene expression changes during mouse preimplantation development. Dev Cell, 6:117-131. 14723852 10.1016/s1534-5807(03)00373-3

[36] Rodríguez-Nuevo A, Torres-Sanchez A, Duran JM, De Guirior C, Martínez-Zamora MA, Böke E (2022). Oocytes maintain ROS-free mitochondrial metabolism by suppressing complex I. Nature, 607:756-761. 35859172 10.1038/s41586-022-04979-5 PMC9329100

[37] Desler C, Hansen TL, Frederiksen JB, Marcker ML, Singh KK, Juel Rasmussen L (2012). Is There a Link between Mitochondrial Reserve Respiratory Capacity and Aging? J Aging Res, 2012:e192503. 10.1155/2012/192503 PMC3375017 22720157

[38] Qi X, Rusch NJ, Fan J, Mora CJ, Xie L, Mu S, et al. (2023). Mitochondrial proton leak in cardiac aging. GeroScience, 45:2135-2143. 36856945 10.1007/s11357-023-00757-x PMC10651624

[39] Wang G, Han T, Nijhawan D, Theodoropoulos P, Naidoo J, Yadavalli S, et al. (2014). P7C3 neuroprotective chemicals function by activating the rate-limiting enzyme in NAD salvage. Cell, 158:1324-1334. 25215490 10.1016/j.cell.2014.07.040 PMC4163014

[40] Di Emidio G, Falone S, Vitti M, D’Alessandro AM, Vento M, Di Pietro C, et al. (2014). SIRT1 signalling protects mouse oocytes against oxidative stress and is deregulated during aging. Hum Reprod, 29:2006-2017. 24963165 10.1093/humrep/deu160

[41] Tatone C, Di Emidio G, Barbonetti A, Carta G, Luciano AM, Falone S, et al. (2018). Sirtuins in gamete biology and reproductive physiology: emerging roles and therapeutic potential in female and male infertility. Hum Reprod Update, 24:267-289. 29447380 10.1093/humupd/dmy003

[42] Tatone C, Di Emidio G, Vitti M, Di Carlo M, Santini S, D’Alessandro AM, et al. (2015). Sirtuin Functions in Female Fertility: Possible Role in Oxidative Stress and Aging. Oxid Med Cell Longev, 2015:659687. 26075037 10.1155/2015/659687 PMC4436464

[43] Hearne A, Chen H, Monarchino A, Wiseman JS (2020). Oligomycin-induced proton uncoupling. Toxicol In Vitro, 67:104907. 32502624 10.1016/j.tiv.2020.104907

[44] Scott R, Zhang M, Seli E (2018). Metabolism of the oocyte and the preimplantation embryo: implications for assisted reproduction. Curr Opin Obstet Gynecol, 30:163-170. 29708901 10.1097/GCO.0000000000000455

[45] Zimmermann H (2021). History of ectonucleotidases and their role in purinergic signaling. Biochem Pharmacol, 187:114322. 33161020 10.1016/j.bcp.2020.114322

[46] Linden J, Koch-Nolte F, Dahl G (2019). Purine Release, Metabolism, and Signaling in the Inflammatory Response. Annu Rev Immunol, 37:325-347. 30676821 10.1146/annurev-immunol-051116-052406

[47] Camacho-Pereira J, Tarragó MG, Chini CCS, Nin V, Escande C, Warner GM, et al. (2016). CD38 dictates age-related NAD decline and mitochondrial dysfunction through a SIRT3-dependent mechanism. Cell Metab, 23:1127-1139. 27304511 10.1016/j.cmet.2016.05.006 PMC4911708

[48] Di Emidio G, Falone S, Artini PG, Amicarelli F, D’Alessandro AM, Tatone C (2021). Mitochondrial Sirtuins in Reproduction. Antioxidants (Basel), 10:1047. 34209765 10.3390/antiox10071047 PMC8300669

