# Supplementary material for: NAD+ Metabolism and Mitochondrial Activity in the Aged Oocyte: Focus on the Effects of NAMPT Stimulation
Source: Aging Dis. 2024 Sep 6;15(6):2828–51. doi: 10.14336/AD.2024.0241 (PMC11567263; doi:10.14336/AD.2024.0241)
Supplement: Supplementary file 1 — The Supplementary data can be found online at: www.aginganddisease.org/EN/10.14336/AD.2024.0241. [file AD-15-6-2828-s.pdf]

SUPPLEMENTARY DATA

NAD$^+$ Metabolism and Mitochondrial Activity in the Aged Oocyte: Focus on the Effects of NAMPT Stimulation

Giovanna Di Emidio, Teresa Vergara, Fani Konstantinidou, Irene Flati, Liborio Stuppia, Paolo Giovanni Artini, Valentina Gatta, Stefano Falone, Carla Tatone

© 2024. Di Emidio G et al. Published online at http://www.aginganddisease.org/EN/10.14336/AD.2024.0241

SUPPLEMENTARY DATA

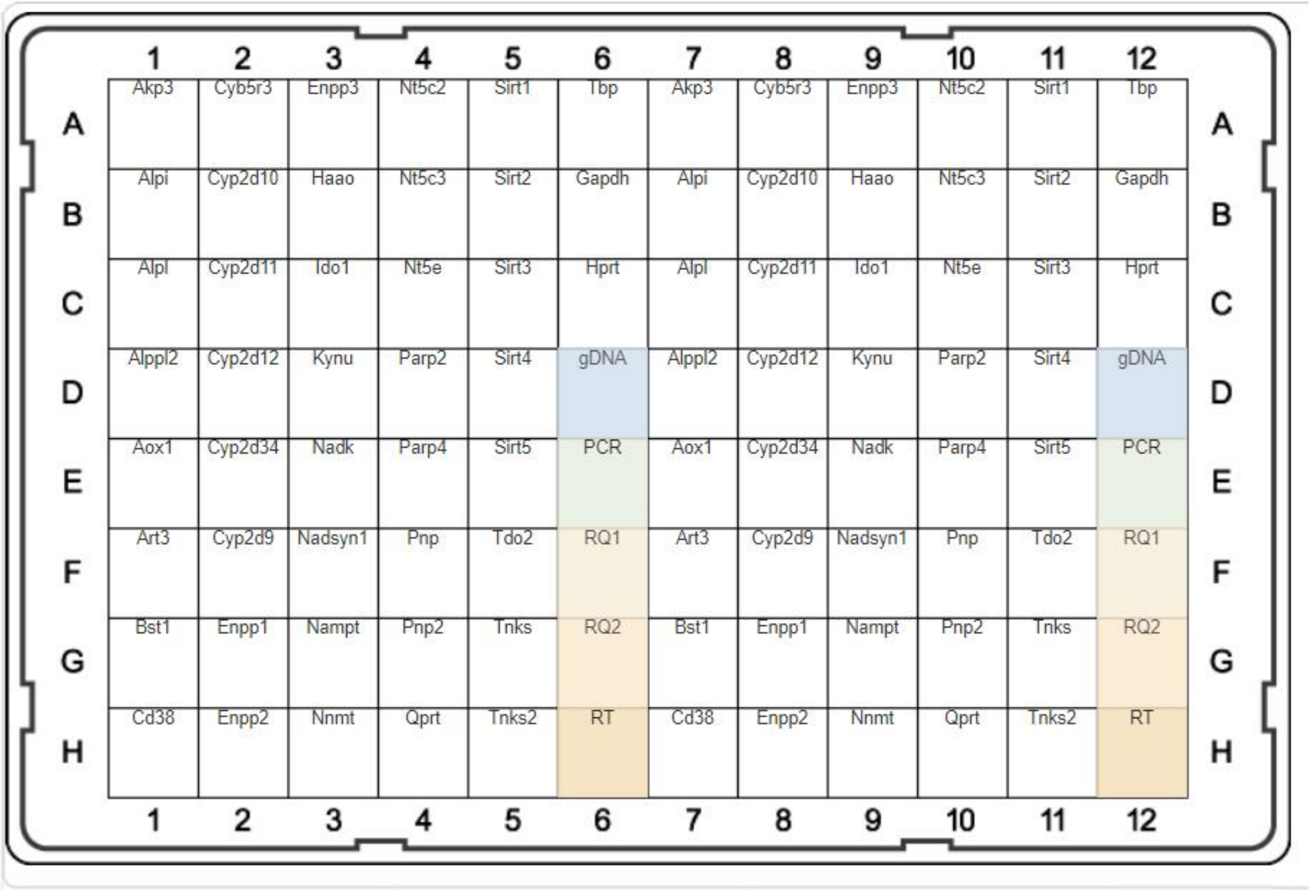

Supplementary Figure 1. Plate scheme of targets and controls contained in the NAD Metabolism M96 Predesigned Array (Bio-Rad Laboratories, CA, USA). Akp3: Intestinal Alkaline Phosphatase 3; Alpi: Intestinal Alkaline Phosphatase; Alpl: Alkaline Phosphatase, Biomineralization Associated; Alppl2: Alkaline Phosphatase, Placental Like 2; Aox1: Aldehyde Oxidase 1; Art3: ADP-Ribosyltransferase 3; Bst1: Bone Marrow Stromal Cell Antigen 1; Cd38: CD38 Molecule (2'-Phospho-Cyclic-ADP-Ribose Transferase); Cyb5r3: Cytochrome B5 Reductase 3; Cyp2d9: Cytochrome P450 Family 2 Subfamily D Member 9; Cyp2d10: Cytochrome P450 Family 2 Subfamily D Member 10; Cyp2d11: Cytochrome P450 Family 2 Subfamily D Member 11; Cyp2d12: Cytochrome P450 Family 2 Subfamily D Member 12; Cyp2d34: Cytochrome P450 Family 2 Subfamily D Member 34; Enpp1: Ectonucleotide Pyrophosphatase/Phosphodiesterase 1; Enpp2: Ectonucleotide Pyrophosphatase/Phosphodiesterase 2; Enpp3: Ectonucleotide Pyrophosphatase/Phosphodiesterase 3; Gapdh: Glyceraldehyde-3-Phosphate Dehydrogenase; Haao: 3-Hydroxyanthranilate 3,4-Dioxygenase; Hprt: Hypoxanthine Phosphoribosyltransferase; Ido1: Indoleamine 2,3-Dioxygenase 1; Kynu: Kynureninase; Nadk: NAD Kinase; Nadsyn1: NAD Synthetase 1; Nampt: Nicotinamide Phosphoribosyltransferase; Nnmt: Nicotinamide N-Methyltransferase; Nt5c2: 5'-Nucleotidase, Cytosolic II; Nt5c3: 5'-Nucleotidase, Cytosolic III; Nt5e: 5'-Nucleotidase Ecto; Parp2: Poly(ADP-Ribose) Polymerase 2; Parp4: Poly(ADP-Ribose) Polymerase Family Member 4; Pnp: Purine Nucleoside Phosphorylase; Pnp2: Purine Nucleoside Phosphorylase 2; Qprt: Quinolinate Phosphoribosyltransferase; Sirt1: Sirtuin 1; Sirt2: Sirtuin 2; Sirt3: Sirtuin 3; Sirt4: Sirtuin 4; Sirt5: Sirtuin5; Tbp: TATA-Box Binding Protein; Tdo2: Tryptophan 2,3-Dioxygenase; Tnks: Tankyrase; Tnks2: Tankyrase2.

© 2024. Di Emidio G et al. Published online at http://www.aginganddisease.org/EN/10.14336/AD.2024.0241

SUPPLEMENTARY DATA

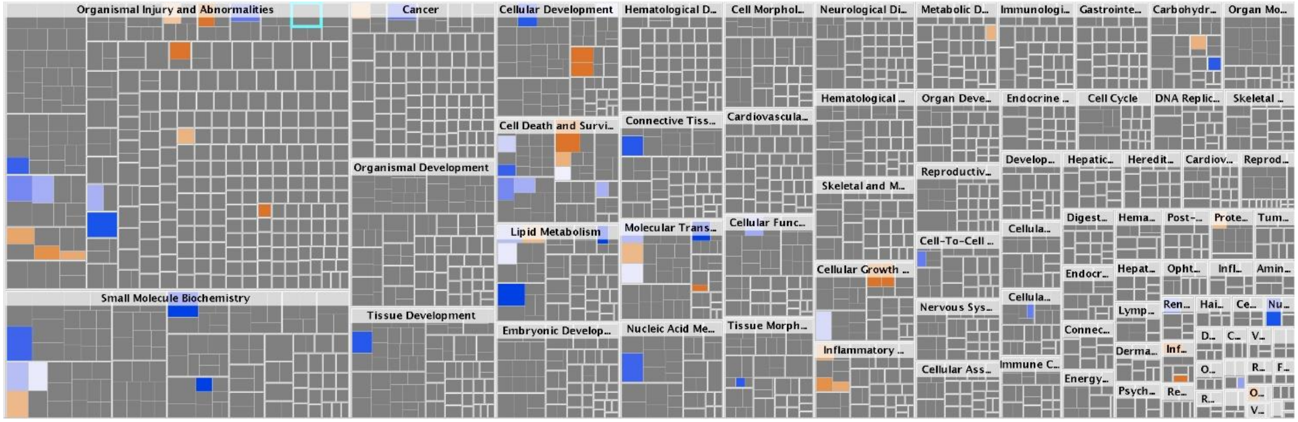

Supplementary Figure 2. Box plots of the whole set of functions regulated by our dataset in aged oocytes. Each box represents a biological class, which is subdivided into secondary functions. The size of the box depends on the significant IPA-inferred p-values. The activated functions are represented in orange, while the inhibited ones in blue. The color is determined by IPA's z-score. z-scores greater than 2 or smaller than -2 may be considered significant.

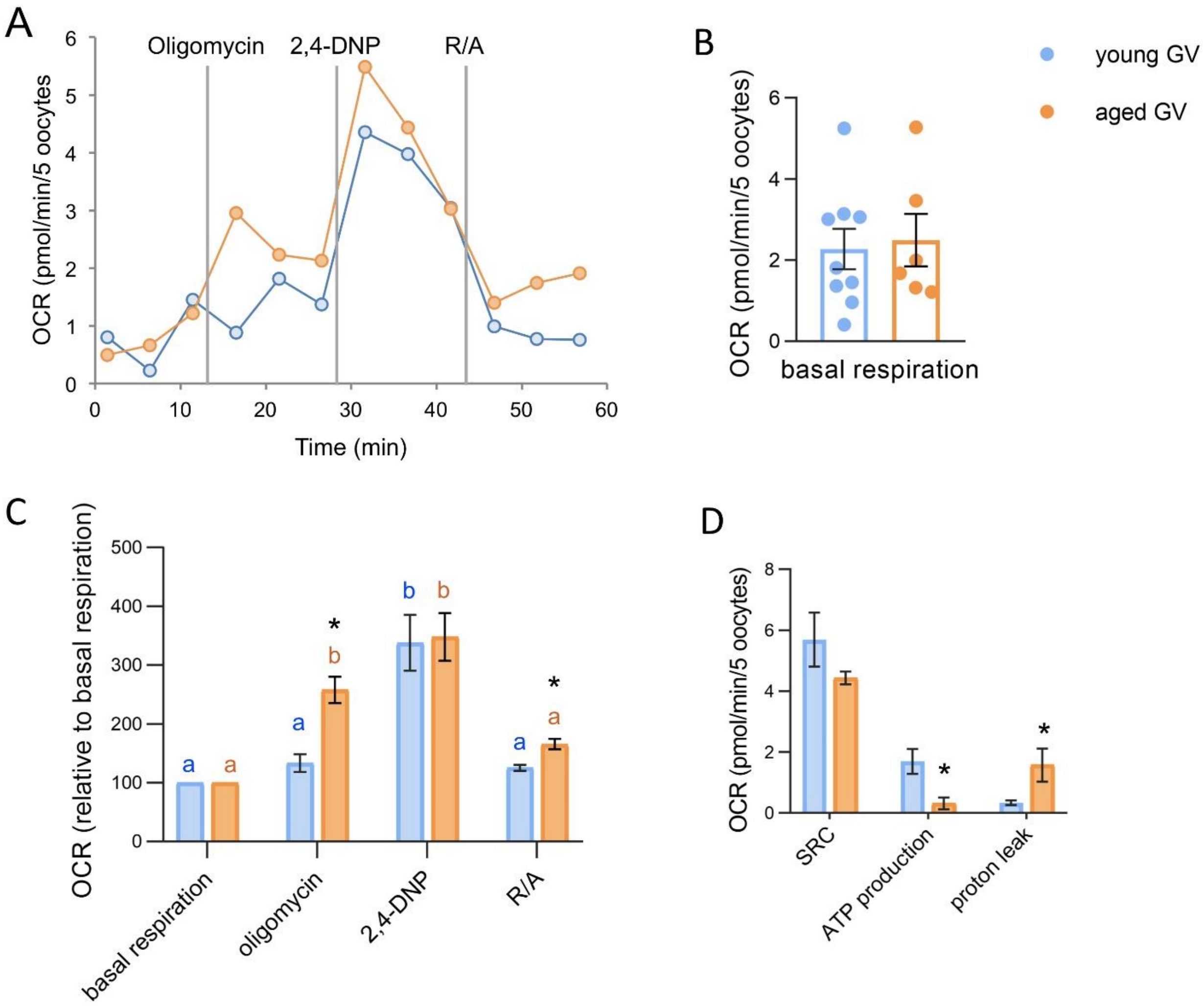

© 2024. Di Emidio G et al. Published online at http://www.aginganddisease.org/EN/10.14336/AD.2024.0241

SUPPLEMENTARY DATA

Supplementary Figure 3. Bioenergetic profile of young and aged GV oocytes. (**A**) Representative profile of live measurements of changes OCR of young and aged GV oocytes upon injection of mitochondrial inhibitors. (**B**) Basal respiration (mean of third measure) of young and aged GV oocytes. Pools of 5-8 oocytes from 3-6 mice were measured. In brackets numbers of pools: young (n=9); aged (n=6). Statistical analysis by unpaired t-test: not significant. (**C**) Mean values of OCR after oligomycin, 2,4-DNP and R/A of young and aged MII oocytes. Pools of 5-8 oocytes from 3-6 mice were measured. In brackets numbers of pools: young (n=4); aged (n=3). Oocyte response to addition of mitochondrial inhibitors was analyzed by one way ANOVA, followed by Student-Newman-Keuls multiple comparison. Different letters indicate a $p<0.05$ in young (blue) and aged (orange) MII oocytes. *$p<0.05$ indicates differences in OCR between young and aged MII oocytes after t-test analysis. (**D**) Spare respiratory capacity (SRC), ATP production and proton leak obtained from live measurements of OCR. Statistical analysis by unpaired t-test for SRC (not significant) and for proton leak ($p=0.016$); or by Mann Whitney test *$p=0.026$ for ATP production.

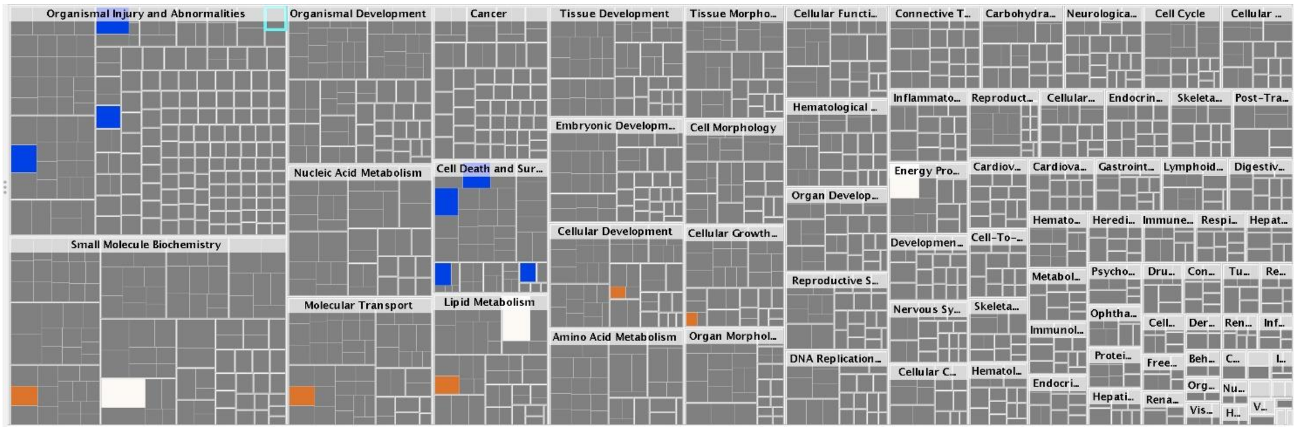

Supplementary Figure 4. Box plots of the whole set of functions regulated by our dataset in aged oocytes exposed to P7C3. Each box represents a biological class, which is subdivided into secondary functions. The size of the box depends on the significant IPA-inferred p-values. The activated functions are represented in orange, while the inhibited ones in blue. The color is determined by IPA’s z-score. z-scores greater than 2 or smaller than -2 may be considered significant.

© 2024. Di Emidio G et al. Published online at http://www.aginganddisease.org/EN/10.14336/AD.2024.0241

SUPPLEMENTARY DATA

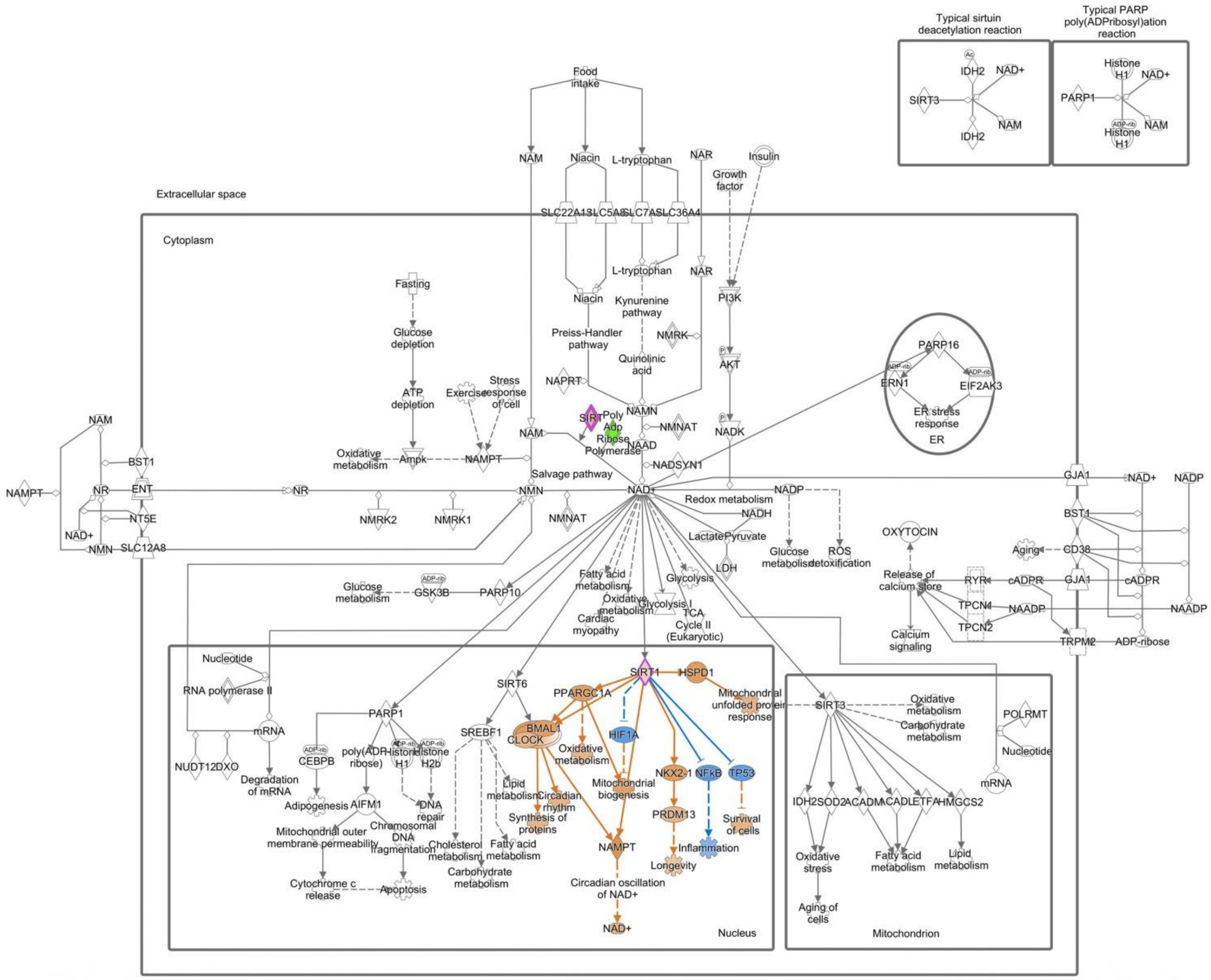

Supplementary Figure 5. Network diagram highlighting the effects of NAMPT stimulation on NAD$^+$ metabolism in the aged IVM oocyte. In the nucleus, the increased levels of SIRT1 detected from dataset leads to predicted activation of mitochondrial unfolded protein response, oxidative metabolism, mitochondrial biogenesis, and predicted a NAD$^+$ increase. NADD: nicotinic acid adenine dinucleotide; NAADP: nicotinic acid adenine dinucleotide phosphate; NAD: nicotinamide adenine dinucleotide; NADP: nicotinamide adenine dinucleotide phosphate; NAM: niacinamide/nicotinamide; NAMN: nicotinic acid d-ribonucleotide; NAR: nicotinic acid d-ribonucleoside; NMN: nicotinamide mononucleotide; NR: nicotinamide ribonucleoside

© 2024. Di Emidio G et al. Published online at http://www.aginganddisease.org/EN/10.14336/AD.2024.0241

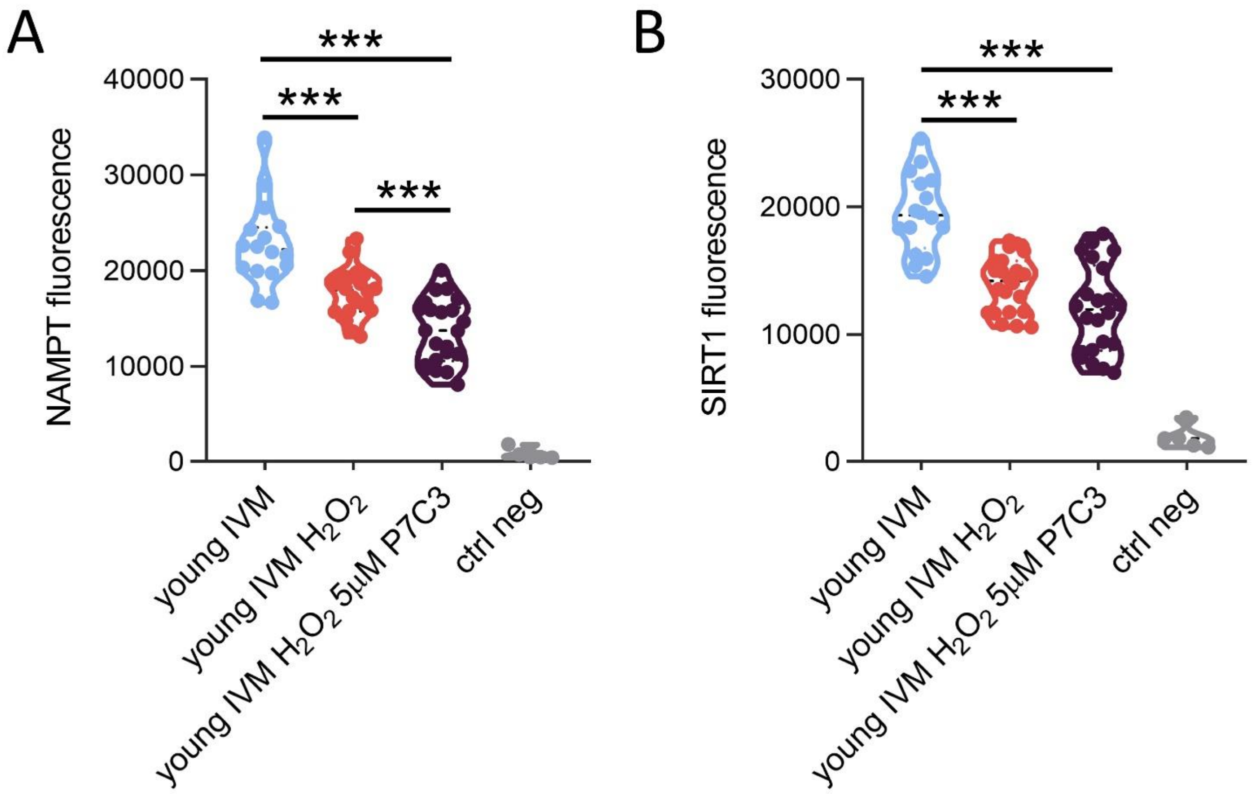

Supplementary Figure 6. Effect of NAMPT stimulation by P7C3 on the protein level of NAMPT and SIRT1, key NAD$^+$ producing and consuming enzymes, of young oocytes exposed to oxidative stress. (**A**) Quantification of fluorescence intensity of NAMPT in young, H_2O_2 or H_2O_2 P7C3 IVM oocytes. 15-25 oocytes isolated from 3-6 animals were analyzed. The experiment was repeated three times. Statistical analysis by one-way ANOVA: $p<0.001$; followed by Tukey's multiple comparisons test: ***$p<0.001$. (**B**) Quantification of fluorescence intensity of SIRT1 in young, H_2O_2 or H_2O_2 IVM oocytes. 15-25 oocytes isolated from 3-6 animals were analyzed. The experiment was repeated three times. Statistical analysis by one-way ANOVA: $p<0.001$; followed by Tukey's multiple comparisons test: ***$p<0.001$.

© 2024. Di Emidio G et al. Published online at http://www.aginganddisease.org/EN/10.14336/AD.2024.0241
